# Halogen Interactions in Halogenated Oxindoles: Crystallographic and Computational Investigations of Intermolecular Interactions

**DOI:** 10.3390/molecules26185487

**Published:** 2021-09-09

**Authors:** Rodrigo A. Lemos Silva, Demetrio A. da Silva Filho, Megan E. Moberg, Ted M. Pappenfus, Daron E. Janzen

**Affiliations:** 1 Institute of Physics, University of Brasilia, Brasilia 70910-900, Brazil; silvarodrigo021@gmail.com (R.A.L.S.); dasf@unb.br (D.A.d.S.F.); 2 International Center for Condensed Matter Physics, University of Brasilia, CP 04455, Brasilia 70919-970, Brazil; 3 Department of Chemistry & Biochemistry, St. Catherine University, St. Paul, MN 55105, USA; memoberg681@stkate.edu; 4 Division of Science and Mathematics, University of Minnesota, Morris, MN 56267, USA; pappe001@morris.umn.edu

**Keywords:** X-ray crystal structure, halogen interactions, QTAIM, NBO

## Abstract

X-ray structural determinations and computational studies were used to investigate halogen interactions in two halogenated oxindoles. Comparative analyses of the interaction energy and the interaction properties were carried out for Br···Br, C-H···Br, C-H···O and N-H···O interactions. Employing Møller–Plesset second-order perturbation theory (MP2) and density functional theory (DFT), the basis set superposition error (BSSE) corrected interaction energy (E_int_(BSSE)) was determined using a supramolecular approach. The E_int_(BSSE) results were compared with interaction energies obtained by Quantum Theory of Atoms in Molecules (QTAIM)-based methods. Reduced Density Gradient (RDG), QTAIM and Natural bond orbital (NBO) calculations provided insight into possible pathways for the intermolecular interactions examined. Comparative analysis employing the electron density at the bond critical points (BCP) and molecular electrostatic potential (MEP) showed that the interaction energies and the relative orientations of the monomers in the dimers may in part be understood in light of charge redistribution in these two compounds.

## 1. Introduction

Molecular crystal engineering relies on various types of intermolecular interactions competing to drive solid-state packing. While the strength of hydrogen bonding may dominate packing in structures where possible, the importance of other competing interactions has drawn increasing attention from a supramolecular perspective [1,2]. From C-H···π [3] and π−π [4] to less studied chalcogen bonding [5] and halogen bonding [6], the roles and relative strengths of these previously considered weak interactions are now employed as key features in crystal packing design. Some structural studies and computational work provide evidence that specific halogen–halogen interactions can compete with [7] or exceed the interaction strengths of traditional hydrogen bonding [8,9,10].

Halogen–halogen noncovalent interactions of two primary types have been described. Type I interactions are typically symmetrical and involve dispersion-type interactions. In contrast, Type II are asymmetrical and involve a σ-hole region of one halogen directed at the nucleophilic region of another halogen. Halogen bonding is typically reserved for Type 2 halogen–halogen short contacts. The highly directional nature of these Type 2 contacts has been exploited to direct crystal packing through halogen–halogen interactions [11,12,13]. Halogen–halogen interactions have been studied in detail from both a crystallographic lens [14,15] as well as in theoretical investigations [16,17,18,19]. For instance, a study by Bolotin et al. [20] revealed that even weak type I halogen···halogen interactions, involving Br···Br contacts, could be the driving forces for the crystallization of a primary organic peroxo compound.

Additionally, multivalent halogen bonding has also presented a growing interest among the scientific community [21,22]. In a recent study, Bauzá et al. [23] investigated the importance of substituent effects in a series of multivalent halogen bonding complexes. Among their finds, it can be highlighted that complexes involving electron-withdrawing substituents obtained more favorable binding energy. This electron-withdrawing substituent character was also explored by Janjić et al. [19]. In their study, the replacement of a hydrogen by a fluorine resulted in an increased interaction energy and in the number of multiple interactions of F···F contacts. In related work, Gurbanov and coworkers have examined halogen substituent effects and competition between halogen interactions and halogen···π interactions [24]. Halogen bonding has also been studied in the presence of additional halogen···metal interactions [25] and oxygen···π interactions [26].

This study explores the intermolecular features of two halogenated oxindoles by analysis of X-ray crystallographic data and through detailed computational investigations. In particular, the effects of fluorine substitution were evaluated. These compounds were chosen as oxindoles are of particular interest for their use in pharmaceutical [27,28] and organic material applications [29,30]. The halogenated oxindoles of this study possess an ensemble of potential competing intermolecular interactions including hydrogen-bonding and several types of halogen interactions. As heavier halogen substituents in pharmaceuticals have gained interest for their potential to enhance affinity protein–ligand complexes [31], and introduction of fluorine atoms is a common strategy to enhance hydrophobicity of therapeutic targets, a better understanding of competition between halogen interactions and other interactions is needed. X-ray structures of these oxindoles are analyzed for differences in intra- and intermolecular features including packing. Computational investigations were then carried out to explore interaction energies of supramolecular dimers, Quantum Theory of Atoms in Molecules (QTAIM) calculations provided insight on contact energies, and Natural Bond Orbital (NBO) analysis provided donor–acceptor orbital interaction pathways. Molecular electrostatic potential (MEP) maps were also examined to help rationalize features of the intermolecular interactions analyzed.

## 2. Results and Discussion

### 2.1. X-ray Structures of 6-Bromooxindole (**1**) and 6-Bromo-4-fluoro-indolin-2-one (**2**)

**Figure 1:**
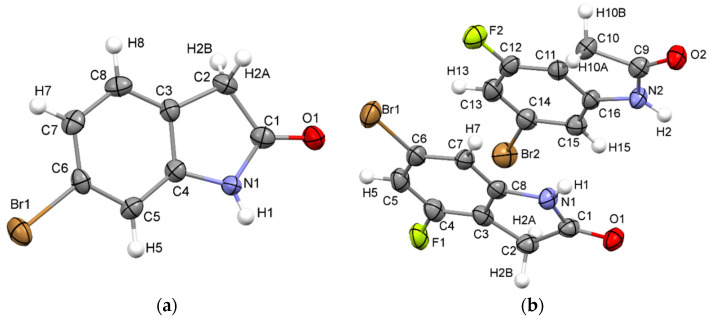
Numbered thermal ellipsoid diagrams (50% probability) of structure **1** (**a**) and structure **2** (**b**).

Single-crystal X-ray structures were collected for compounds **1** and **2**. Data collection and refinement details are reported in Table 1. The asymmetric unit of each structure is shown in Figure 1. The asymmetric unit of **1** consists of a single complete molecule with all atoms on general positions, while structure **2** includes two complete unique molecules on general positions. The two independent molecules of structure **2** do not exhibit significant geometric differences as the RMS deviation of an overlay of the coordinates of the two molecules non-hydrogen atoms is only 0.0248. A close look at intramolecular features of **1** and **2** shows a small contraction of all analogous bond lengths in **2** compared with **1** (Figure S1). All bond lengths in both structures fall in typical ranges but introducing the highly electronegative fluorine substituent in **2** likely contributes to these bond length contractions.

While not isomorphous, the packing of **1** and **2** can both be described as herringbone arrangements of infinite π-stacks (Figure S2). Structure **1** has a single repeating parallel nearest interplanar distance of 3.38 Å. The stacking vector in **1** is parallel to the crystallographic *a* axis. In contrast, structure **2** has π-stacks composed of unique alternating molecules of the asymmetric unit in a nearly parallel fashion (angle between molecular least-squares planes = 0.76°) with a nearest average interplanar separation of 3.35Å. The stacking vector in **2** is parallel to the crystallographic *a* axis. In both structures, π-stacks engage with the nearest π-stacks via dimeric intermolecular hydrogen bonding interactions. However, in the structure of **1**, short Br···Br contacts can be found between further neighboring π-stacks. The structure of **1** is isomorphous with the previously reported chlorinated analog 6-chloro-1,3-dihydro-2H-indol-2-one [32]. 

Examination of the short atomic intermolecular features of **1** and **2** reveals similar hydrogen-bonding motifs but unique interactions involving the halogen atom contacts. Details of short intermolecular contacts can be found in Table 2.

**Figure 2:**
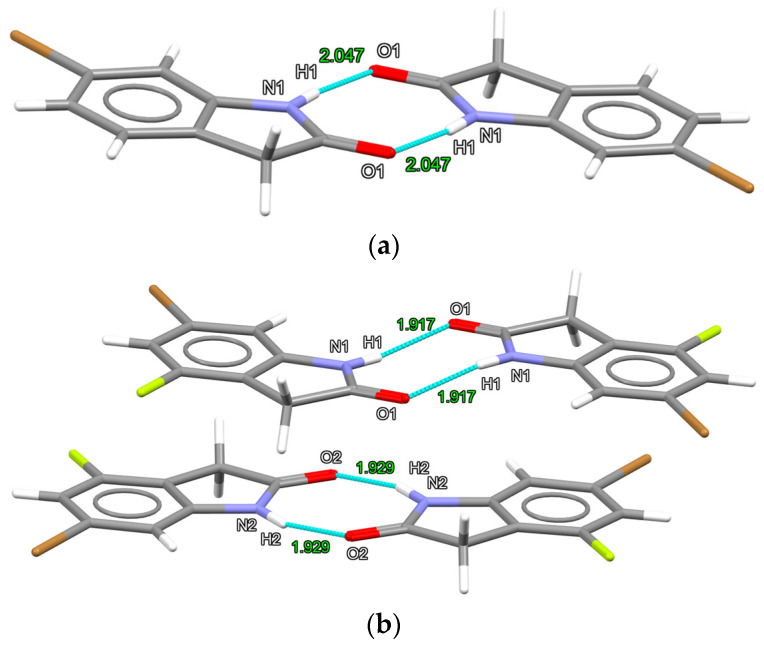
R2,2(8) hydrogen bonding dimers in structure **1** (**a**) and structure **2** (**b**). Intermolecular atom distances in units of Å.

Both structures exhibit a dimeric intermolecular hydrogen-bonding pattern involving the hydrogen donor amide N-H with the amide oxygen as hydrogen bond acceptor in a R 2,2(8) graph set motif located over a crystallographic inversion center. (Figure 2).

**Figure 3:**
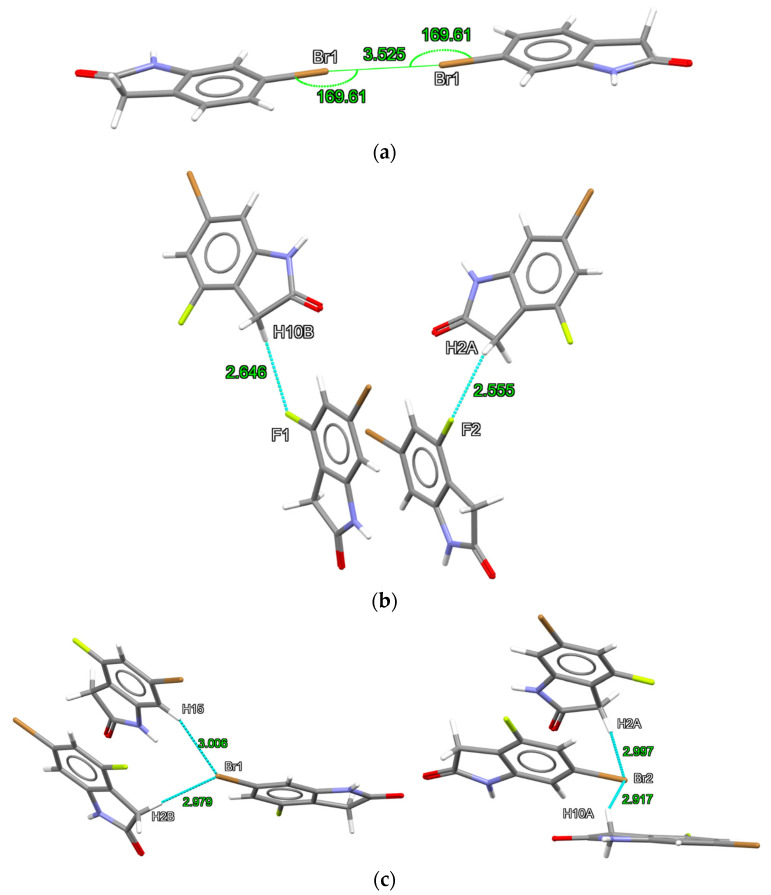
Short intermolecular contacts involving halogens in **1** (**a**) (Br···Br), **2** (**b**) (C-H···F), and **2** (**c**) (C-H···Br). Intermolecular atom···atom distances in units of Å, angles in degrees.

The intermolecular nitrogen···oxygen distances in structure **2** are shorter (2.771(4) Å, 2.760(4) Å) than for structure **1** (2.843(6) Å). The chlorinated analog of **1** exhibits hydrogen bonding N···O distances very similar to those of **1** (2.840(2) Å). Short Br···Br contacts are present in **1** exhibiting a type I geometry (θ_1_ = θ_2_ = 169.6(1)) with a distance of 3.525 Å (distance—sum of Van der Walls radii = −0.175 Å). (Figure 3) The same type I of halogen···halogen contacts are found in the chlorinated analog of **1** ((θ_1_ = θ_2_ = 170.87(5)°; Cl···Cl distance = 3.438 Å; distance—sum of vdW radii = −0.062 Å) [32]. This is consistent with previous observations that halogen···halogen contacts result in weaker interactions (using vdW radii sum distance differences as a proxy) in the order of decreasing strength I > Br > Cl >> F. Structure **2** is absent of short Br···Br intermolecular contacts (as well as F···F and Br···F contacts), but now includes C-H···F and C-H···Br contacts less than van der Waals radii sums (Figure 3).

Each fluorine of **2** has a short intermolecular contact with a methylene C-H. Each bromine of **2** has two short contacts, one with two unique methylene C-H and the other with one methylene C-H and one aromatic C-H. These new interactions may result from increased ability of the methylene hydrogens to act as C-H donors induced by the electron-withdrawing nature of the β carbon fluorination. Aromatic and methylene C-H···O contacts are present in **1** and **2** but this C-H···O interaction in **2** is paired with the methylene C-H···F interaction in an R 2,2(8) motif (Figure S3).

### 2.2. Computational Studies of Structures **1** and **2**

#### 2.2.1. QTAIM Analysis of Supramolecular Dimers of **1** and **2**

While short interatomic distances and analysis of packing features’ geometry imply the presence of intermolecular interactions, that are engaged to drive crystal packing, computational methods were employed to estimate the relative contributions of these identified intermolecular interactions. The most obvious intermolecular feature, hydrogen bonding interactions, is retained in both structures **1** and **2**, while the presence of fluorine disrupts halogen–halogen interactions only found in **1** and also engages C-H···halogen interactions. We analyzed the relative strengths of the intermolecular interactions in **1** and **2** by performing calculations involving coordinates of dimer assemblies taken from our crystallographic data. Dimer supramolecular assemblies were chosen based on the presence of atom···atom contacts less than vdW radii sums. The cartesian coordinates of the dimers are provided in Table S4. Hydrogen bonding dimers were investigated for structures **1** (1-NHONHO) and **2** (2a-NHONHO and 2b-NHONHO, one for each crystallographically independent molecule). A dimer with a unique C-H···Br interaction of **2** was examined (2-CHBrBr). Dimers with C-H···O interactions were also investigated (1-CHOCHBr, 2a-CHOCHF, 2b-CHOCHF). To investigate details of the hydrogen bonding, halogen–halogen, C-H···halogen, and C-H···O interactions in these structures, QTAIM methods and reduced density gradient (RDG) index analysis were utilized at the MP2/def2-TZVP theory level. QTAIM parameters using the MP2/def2-TZVP theory level are shown in Table 3. 

**Figure 4:**
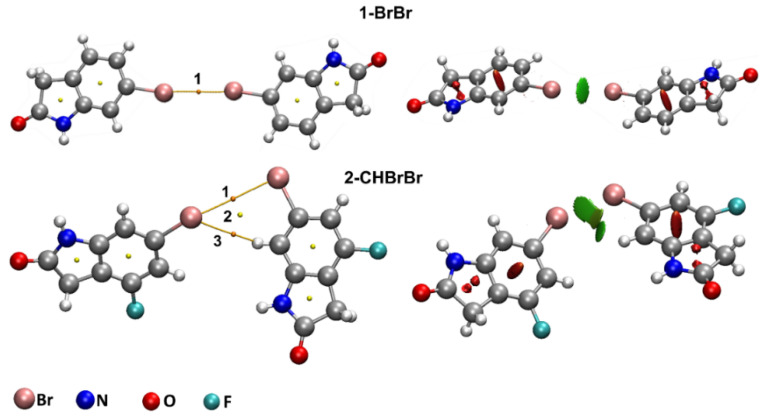
Critical points and RDG isosurfaces for 1-BrBr and 2-CHBrBr dimers obtained with the MP2/def2-TZVP theory level. The orange points indicate bond critical points (BCP), yellow points indicate ring critical points (RCP) and yellow lines indicate the bond paths. The RDG isosurfaces where obtained with isovalues of 0.65 a.u. The green-colored region indicates a van der Waals interaction in the Br···Br contact regions.

**Figure 5:**
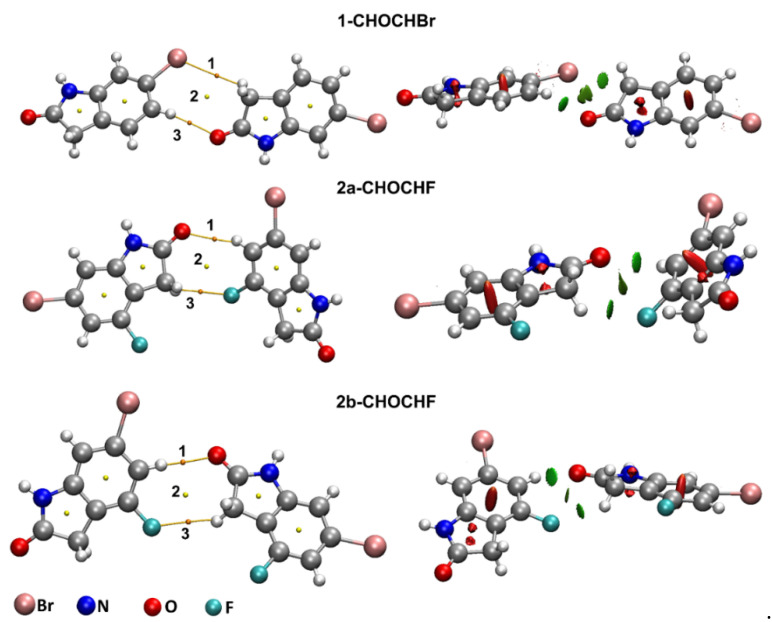
Critical points and RDG isosurfaces for 1-CHOCHBr, 2a-CHOCHF and 2b-CHOCHF dimers obtained with MP2/def2-TZVP theory level. The orange points indicate bond critical points (BCP), yellow points indicate ring critical points (RCP) and yellow lines indicate the bond paths. The RDG isosurfaces where obtained with isovalues of 0.65 a.u. The green-colored region indicates a van der Waals interaction in Br···H, O···H and F···H contact regions.

**Figure 6:**
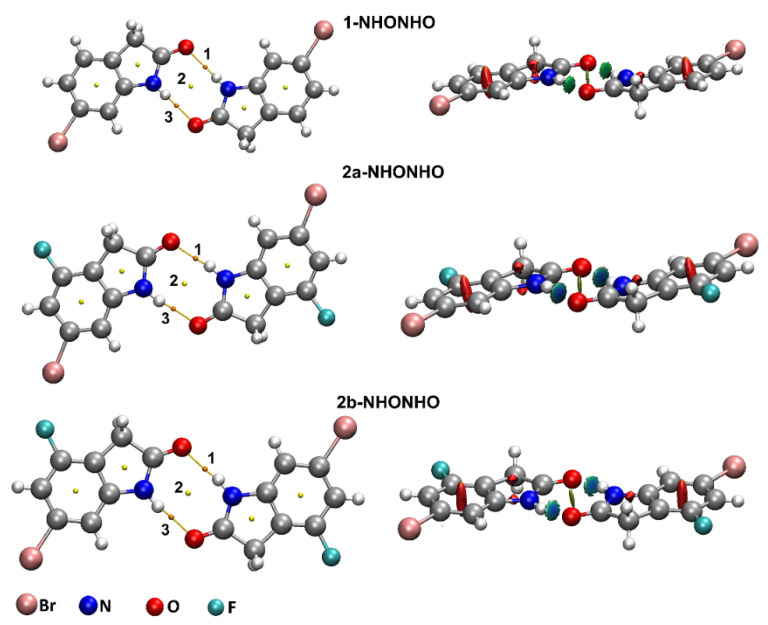
Critical points and RDG isosurfaces for 1-NHONHO, 2a-NHONHO and 2b-NHONHO dimers obtained with MP2/def2-TZVP theory level. The orange points indicate bond critical points (BCP), yellow points indicate ring critical points (RCP) and yellow lines indicate the bond paths. The RDG isosurfaces were obtained with isovalues of 0.65 a.u. The green blue-colored region indicates a hydrogen bonding NH···O interaction.

**Figure 7:**
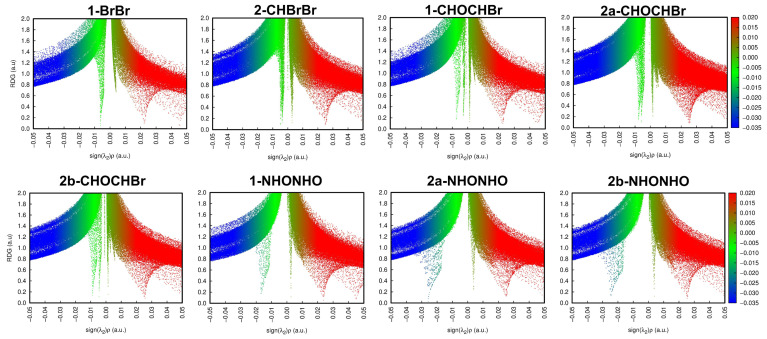
Scatter graph of RDG. Points in the blue region, where $\mathrm{sign}\left(\lambda_{2}\right)\rho$ assumes negative values, are indicative of strong attractive interactions such as a hydrogen bond. Points in the green region, for values of $\mathrm{sign}\left(\lambda_{2}\right)\rho$ close to zero, are indicative of van der Waals contacts. Points in the red region, where $\mathrm{sign}\left(\lambda_{2}\right)\rho$ assumes positive values, are indicative of repulsive effects. These results were obtained with the MP2/def2-TZVP theory level.

With the interest of confirming the tendencies presented by the first method, we also employed the ωB97X-D/def2-TZVP theory level (results in Table S1). Each dimer investigated presented at least one bond critical point (BCP) and bond paths, which are represented, respectively, by orange points and yellow lines in Figure 4, Figure 5 and Figure 6. According to the QTAIM scheme, the presence of bond paths and BCP are evidence of interaction [33,34]. The RDG analysis is presented in the sequence of Figure 4, Figure 5 and Figure 6 and in the scatter graph in Figure 7. The RDG analysis is directly correlated to the QTAIM critical points and provides a visual support in the inter-molecular interaction identification. In this Figure 4, Figure 5, Figure 6 and Figure 7, the green-colored regions are indicative of van der Waals interactions. Regions with blue-colored character indicate possible hydrogen interactions while the red-colored regions are corelated with steric effects. A more detailed color addressment to the interaction types can be view in the subtitles of Figure 4, Figure 5, Figure 6 and Figure 7. 

A Br···Br interaction dimer was investigated for structures **1** (1-BrBr) and **2** (2-CHBrBr). Although no short Br···Br contacts are present in **2**, bond critical points (BCPs) were observed in the theoretical calculations. For the BCPs observed in the Br···Br contacts in 1-BrBr and 2-CHBrBr, as can be observed in Table 3 and Table S1, in both theory levels, the electron density $(\rho_{BCP}$) falls in the range of $10^{-3}\;e/a_{0}^{3}$, the Laplacian of the electron density (∇^2^ρ_BCP_) and the total electron energy density ($H_{BCP}$) are positive. These QTAIM parameters indicate that the Br···Br interactions are closed-shell interactions [33,34,35]. As $\left|G_{BCP}/V_{BCP}\right|>1$, the Br···Br interactions are completely non-covalent interactions [36]. The negative sign of $\lambda_{2}$ indicates an attractive interaction for Br···Br contacts. For the RDG analysis, these Br···Br interactions are observed as green-colored isosurfaces, in Figure 4 and Figure S4, and at $\mathrm{sign}\left(\lambda_{2}\right)\rho \;≅\;0$ values in the scatter graphs presented in Figure 7 and Figure S7. Thus, the QTAIM parameter values together to the RDG analysis, for both theoretical levels, indicate the van der Waals character of this interaction. The values obtained for Br···Br at BCPs presented here are consistent for non-covalent interactions involving Br atoms obtained in previous studies [20,37,38]. 

A BCP for C-H···Br contact was also observed in 2-CHBrBr and 1-CHOCHBr complexes. As shown in Table 3 and Table S1, this BCP also presented QTAIM parameters very similar to those observed for Br···Br contacts, which indicates a vdW attractive interaction. This fact was corroborated by the RDG investigations presented in isosurfaces of Figure 4 and Figure 5, Figures S4 and S5 and by the scatter graph showed in Figure 7 and Figure S7. The QTAIM analysis indicates that the C-H···Br interactions play an essential role in the energetic stabilization of these complexes, especially for 2-CHBrBr. 

The 1-CHOCHBr and 2a/2b-CHOCHF also presented C-H···O interactions. For the 2a/2b-CHOCHF dimers, C-F···H contact was paired with a C-H···O interaction. Each one of these interactions presented BCPs with $\rho_{BCP}$ value close to $10^{-3}\;e/a_{0}^{3}$. As ∇^2^ρ_BCP_ and $H_{BCP}$ are positive and $\left|G_{BCP}/V_{BCP}\right|>1$, with $\lambda_{2}$ negative, the C-H···O and C-F···H interactions can be described as vdW interactions. In fact, from the results presented in Table 3 and Table S1, one can see that the C-H···O and C-F···H contacts presents QTAIM parameters very similar to the Br···Br and Br···C-H contacts, however with a high electron density value at BCPs, especially for C-H···O. Additionally, for 1-CHOCHBr and 2a/2b-CHOCHF, the C-H···O interactions presented a high electron density which would indicate that these are the dominant interactions in these dimers. The RDG green-colored isosurfaces representation are presented in Figure 5 and Figure S5 while, the scatter graphs, with $\mathrm{sign}\left(\lambda_{2}\right)\rho \;\approx 0,\;$ are presented by Figure 7 and Figure S7.

In the BCPs observed in the N-H···O interactions, for 1/2a/2b-NHONHO, in both theory levels, the $\rho_{BCP}$ values are close to $10^{-2}\;e/a_{0}^{3}$, ∇^2^ρ_BCP_ together with $H_{BCP}$ which are positive, and $\left|G_{BCP}/V_{BCP}\right|>1$. The negative values of $\lambda_{2}$ indicate an attractive interaction in the N-H···O contact. These QTAIM parameters are good indicators of closed-shell non-covalent interactions [33,34,35,36]. Taking in count the values for the QTAIM parameters obtained at the BCPs 1 and 3, shown in Table 3 and Table S1, the N-H···O interactions are described as hydrogen bonding. The RDG green blue-colored isosurfaces, in Figure 6, together with the values of $\mathrm{sign}\left(\lambda_{2}\right)\rho$ presented in Figure 7 and Figure S7, corroborate this observation.

The topological parameters obtained here are consistent with hydrogen bond interactions observed in previous studies [39,40]. The larger hydrogen-bonding interaction energies calculated for **2** $\left(E_{int}\left(BSSE\right)=-11.782\frac{\mathrm{kcal}}{\mathrm{mol}}\right)$ vs. **1** ($E_{int}\left(BSSE\right)=-11.465\;\mathrm{kcal}/\mathrm{mol}$) are also consistent with the shorter intermolecular distances observed in the crystal structures.

#### 2.2.2. Natural Bond Orbital Analysis of Supramolecular Dimers of **1** and **2**

**Figure 8:**
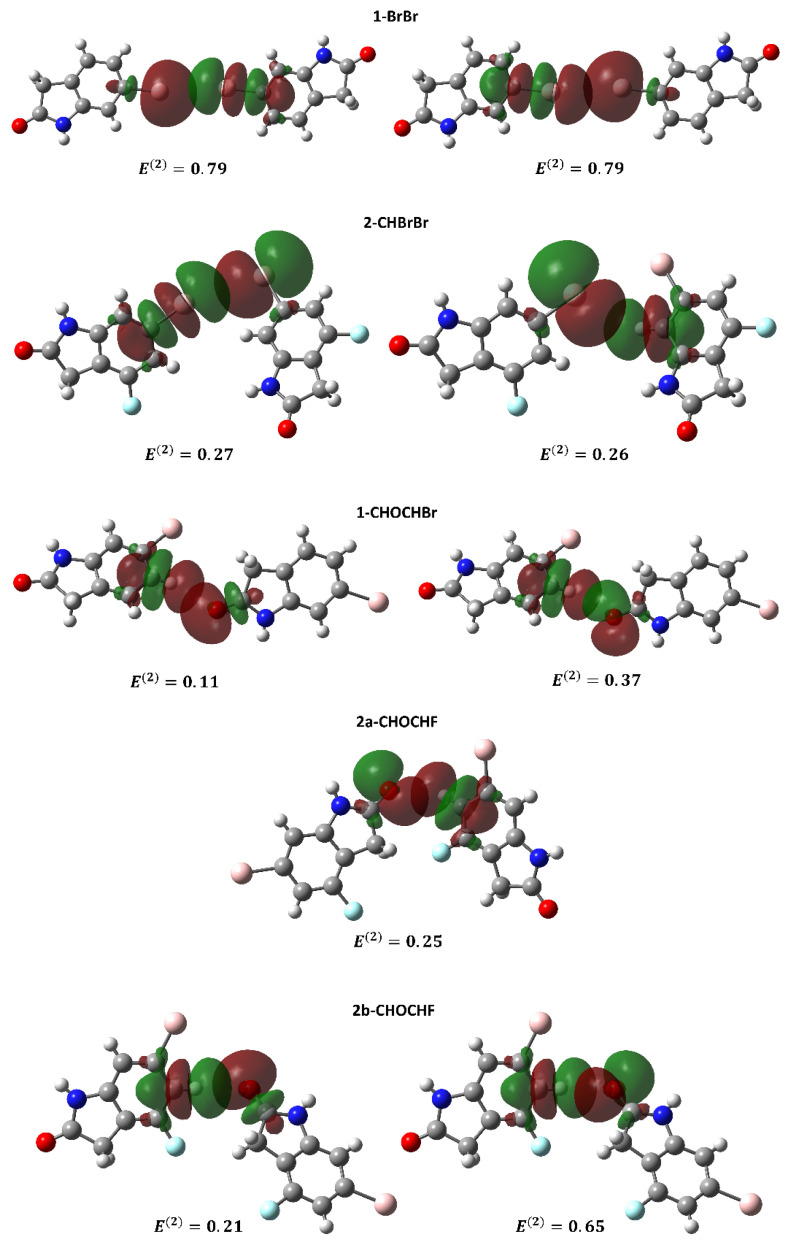

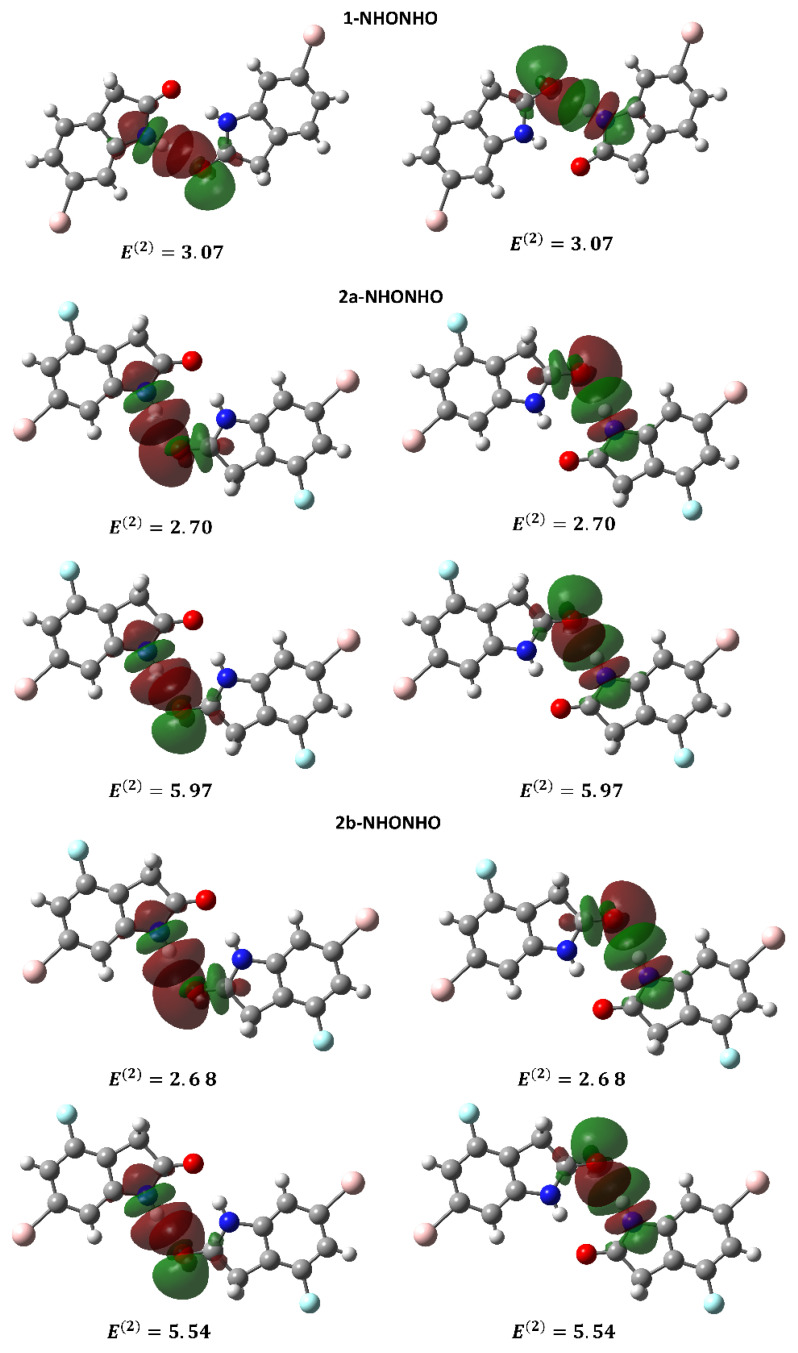
NBO donor and acceptor orbitals for dimers of **1** and **2** obtained with the MP2/def2-TZVP theory level. The NBO second-order energy perturbation $E^{\left(2\right)}$ is given in kcal/mol. The threshold of 0.10 kcal/mol was employed for the NBO orbital printing. Phase relationships between donor and acceptor NBO orbitals are arbitrary.

In order to understand the nature of the orbitals involved in the intermolecular interactions in **1** and **2**, natural bond orbital (NBO) calculations were performed at the MP2/def2-TZVP and ωB97X-D/def2-TZVP theory levels. Table 4 shows the most important donor–acceptor interactions and their second-order perturbation energies $E^{\left(2\right)}$ while, Figure 8 presents these orbitals for all the dimers.

The results presented in Table 4 and Figure 8 are for the MP2/def2-TZVP theory level; the results for ωB97X-D/def2-TZVP are presented in Table S2 and Figure S8. The threshold of 0.1 kcal/mol was adopted for printing values of second-order energy. For 1-BrBr, the main orbital involves a lone-pair (LP) from the Br atom interacting with an anti-bonding (BD*) Br-C orbital. Although the Br···Br contact in 2-CHBrBr does not present a distance smaller than the sum of the Br van der Waals radii, the NBO investigation (as well as the QTAIM analysis) shows a Br···Br interaction and a C-H···Br interaction. These two orbital interactions have similar magnitude $E^{\left(2\right)}$ values. For the Br···Br contact, the NBO contribution involves a lone pair (LP) of Br and a BD* Br-C orbital while for the C-H···Br interaction, the NBO contribution employs a LP of Br and a BD* C-H orbital. In 2-CHBrBr, the geometric relationship of this long intermolecular Br···Br “contact” is more akin to a Type II halogen–halogen interaction (Figure 8 and Figure S8). As the Br···Br distance is beyond vdW contact in 2-CHBrBr, the smaller $E^{\left(2\right)}$ value for the Br···Br contact in 2-CHBrBr compared with 1-BrBr is as expected.

For 1-CHOCHBr and 2a/2b-CHOCHF, the most relevant NBO donor–acceptor interactions occur between a LP of O with a BD* C-H orbital. For 1/2a/2b-NHONHO, all dimers present interactions between an LP of O and a BD* N-H orbital as the most relevant contribution. These results were observed for both theory levels and the pairwise interactions were also corroborated by observations made with QTAIM. The only difference between the two theory levels was that, for the ωB97X-D functional, one more LP O to BD* C-H orbital interaction was observed in the threshold of 0.1 kcal/mol adopted (see Table 4 and Table S2). In addition, the value of the $E^{\left(2\right)}$ energy of the orbital interactions involving 1/2a/2b-NHONHO complexes are higher than the $E^{\left(2\right)}$ values for 1-CHOCHBr, 2a/2b-CHOCHF dimers and for 1-BrBr, 2-CHBrBr systems. 

#### 2.2.3. Interaction Energies and MEP Analysis of Supramolecular Dimers of **1** and **2**

Aiming to verify the energetic stability of these interactions, the interaction energy,$\;E_{int}$, was obtained for dimers of **1** and **2** employing Equation (1). For each dimer, *E_int_*, based on a supramolecular approach and either the $E_{cont}^{HB}$ and $E_{cont}^{a,b,c,d}$, based on the sum of the hydrogen bond energy, *E_HB_*, or halogen energy, *E_XB_*, (depending on the nature of the interaction) were determined employing MP2/def2-TZVP and ωB97X-D/def2-TZVP theory levels. The results for the MP2/def2-TZVP theory level are presented in Table 5 (ωB97X-D/def2-TZVP theory level results in Table S3). Beyond the interaction energy, another quantity usually employed to measure the stability in crystal structure [41,42] is the contact energy,$\;E_{cont}$. As some dimers present more than one interaction and, sometimes with different types, the QTAIM-based contact energies were obtained by summing the different atom pair energies in a dimer. For this purpose, the individual atom–atom contact energy, either halogenic $E_{XB}$ or hydrogenic, $E_{HB}$, was taken into account. $E_{XB}$ was estimated using Tsirelson et al.’s [42], Bauzá et al.’s [18] and Kuznetsov’s [41] procedures, summarized by Equations (2)–(5). For the $\;E_{HB}$ energy, presented in 1/2a/2b-NHONHO dimers, the Espinosa et al. [43] procedure, presented in Equation (6), was employed. The $E_{cont}$ energies were obtained according to the schemes presented by Equations (2)–(11). 

Although $E_{int}$ energies obtained for the complexes using this supramolecular approach are close to $E_{cont}$ for all complexes (the difference between the methods is smaller than the theoretical error), the interaction energy considering basis set superposition error, $E_{int}\left(BSSE\right)$, presented in Table 5, indicates a small difference between contact energy and interaction energy. The exception was observed for 1-BrBr. For this complex, Bauzá et al.’s procedures (Equation (4)) presented an excellent agreement between the $E_{int}\left(BSSE\right)$, obtained by MP2/def2-TZVP and $E_{cont}$ obtained for both theoretical levels. Considering the supramolecular approach, the ωB97X-D/def2-TZVP calculation could not reproduce the attractive interaction energy for 1-BrBr as evidenced by the positive value of $E_{int}\left(BSSE\right)$ obtained by this theoretical calculation presented by Table 3.

In all supramolecular energy interaction calculations and using Tsirelson et al.’s [42], Bauzá et al.’s [18] and Kuznetsov’s [41] procedures, the energetic results indicate a more favorable interaction between the monomers in the 1/2a/2b-NHONHO dimers. This may be due to the hydrogen interactions observed in 1/2a/2b-NHONHO which are stronger than the van der Waals interactions observed in 1-BrBr, 2-CHBrBr, 1-CHOCHBr and 2a/2b-CHOCHF dimers. In addition, the absence of complementary van der Waals interactions in the dimers of 1-BrBr, 2-CHBrBr, 1-CHOCHBr and 2a/2b-CHOCHF is also reflected in their lower values of $E_{int}\left(BSSE\right)$ and $E_{cont}$ when compared to the dimers of 1/2a/2b-NHONHO. The values of $E_{int}\left(BSSE\right)$ obtained here with MP2/def2-TZVP calculations for Br···Br are in agreement with the theoretical values obtained by Capdevila-Cortada et al. [10] for (CBr_4_)_2_ dimer interactions. The order of decreasing strength of interactions for the dimers is as follows: 2b-NHONHO > 2a-NHONHO > 1-NHONHO > 1-CHOCHBr > 2b-CHOCHF > 2a-CHOCHF > 2-CHBrBr > 1-BrBr.

To better understand this interaction order and correlate the electrostatic properties of the molecules studied here to the crystal packing, a molecular electrostatic potential (MEP) map analysis was performed using the crystallographic coordinates for **1** and the two unique molecules of **2** (2a and 2b). Previous work has demonstrated that dominant partial charge concentration, either positive or negative, on specific parts of interacting molecules have important influence in the intermolecular interaction [44,45,46]. Due its high electronegativity, fluorine has the capacity of electron-withdrawing from neighbor atoms [19,47,48] and, for this reason, this atom has become an interesting substituent in many chemical systems in the search for changes the electronic properties or to enhance intermolecular interaction [47,49].

**Figure 9:**
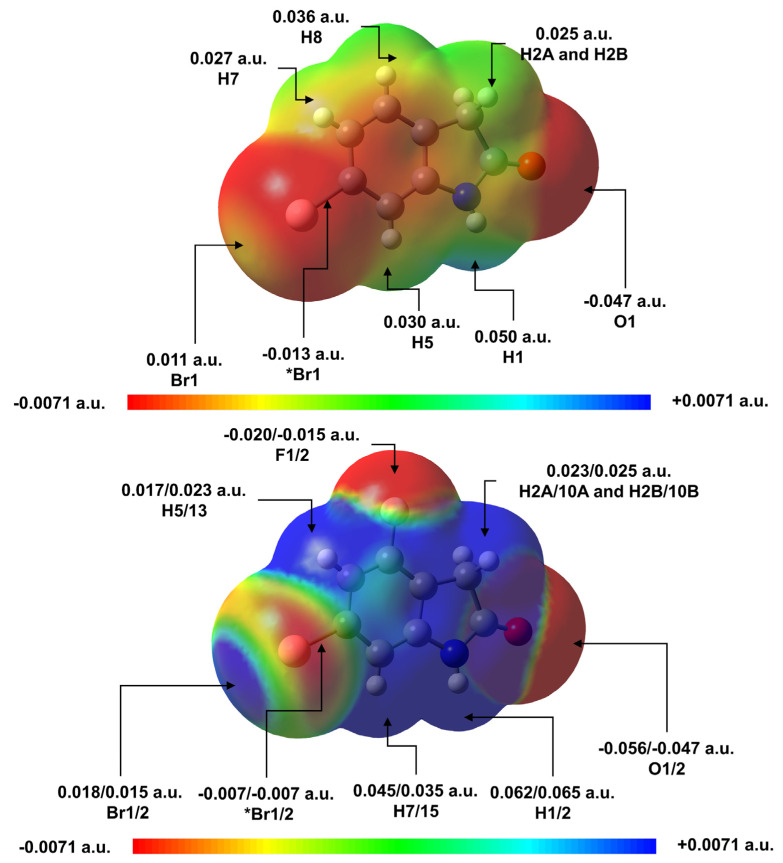
Molecular Electrostatic Potential (MEP) map for **1**, top, and **2a**/**2b**, bottom, calculated with the MP2/def2-TZVP theory level. The arrows indicate the interacting regions of the monomers that formed the dimers presented in Figure 4, Figure 5 and Figure 6.

Through the MEP analysis, shown in Figure 9, the substitution of one hydrogen atom for one fluorine atom produces significant changes in the potential map for **1** vs. **2**. In Figure 9, the interaction regions observed in the crystal structure had the potential values highlighted. The insertion of F1/2 in place of H8 produced a change in the MEP of the entire molecule **1** compared to **2**. This perturbation in the MEP is due to the high electronegativity of the fluorine atom that tends to attract electrons from atoms throughout the molecule [19,47,48]. Changes in potential on Br indicate a more electrophilic behavior in the sigma hole region (highlighted by the increase in the blue region) in **2** vs. **1**. Besides the region around the bromine, in general, all regions of the molecule **2** showed an increase in electrophilic behavior relative to **1**. The increase in electrophilic behavior at these regions favors the interaction of these parts of the molecule with more nucleophilic regions of other molecules. Keeping in mind these changes in the MEP after the addition of the F atom, the molecular interactions between within dimers of **1** and **2** can be better understood and compared with both QTAIM analyses and the $E_{int}$ interaction energy of each dimer.

The increases in the interaction energy of 2-CHBrBr relative to 1-BrBr (Figure 4) may be rationalized with the changes in the MEP maps of **1** and **2**. The increased electrophilicity of hydrogens in **2** (related to the F substitution) is more likely the main contribution of the larger interaction energy than the long Br···Br contact in 2-CHBrBr. The dominance of this H15···Br1 interaction in 2-CHBrBr is confirmed by QTAIM analyses for the two theoretical levels, in which BCP 3, presented in Table 3 and Table S1, showed higher electronic density than BCP 1.

For the 1-CHONHBr, 2b-CHOCHF and 2a-CHOCHF systems, according to the QTAIM parameters, the C-H···O interaction is dominant. This can be explained because of the regions of interactions highlighted in the MEP (Figure 9) where, the interaction between O1···H5 (in 1-CHONHBr) and O1···H15 and O2···H7 (in 2b-CHOCHF and 2a-CHOCHF, respectively) have higher electrostatic potential difference than the interaction between Br1···H2A and H2B or between F1/2···H2A/10A and H2B/1B. It is also observed that the 1-CHONHBr, 2b-CHOCHF and 2a-CHOCHF systems exhibit higher energy stability than the purely halogenic 1-BrBr and 2-CHOBr complexes. This increase in $E_{int}$ may be due to both the increase in interaction pairs and the presence of a C-H···O interaction that is stronger than halogen interactions.

With respect to the 1/2a/2b-NHONHO group of dimers, it is evident that the substitution of the H atom for the F atom favored increased stability of the structures, which can be observed in the increase in $E_{int}$. As can be observed in the MEP difference (Figure 9), the polarity between H1/2···O1/2 increases for the 2a/2b-NHONHO dimers compared with the 1-NHONHO dimer. This fact is reflected in the increased density at BCP 1 and BCP 3 of the 2a/2b-NHONHO complexes with respect to 1-NHONHO dimers. 

## 3. Materials and Methods

Compound **1**, 6-bromooxindole was purchased from TCI and used as received. Compound **2**, 6-bromo-4-fluoro-indolin-2-one, was purchased from Combi-Blocks and used as received.

### 3.1. X-ray Crystallography

Table 1 contains crystal data, collection parameters, and refinement criteria for the crystal structures of 1 and 2. Crystals of **1** and **2** were grown by slow evaporation of saturated solutions in CDCl_3_. Crystals were mounted on the tip of MiTeGen (Ithaca, NY, USA) micromount and X-ray intensity data were measured at 173K using an Oxford Cryosystems (Oxford, UK) desktop cooler with graphite monochromated Mo Kα radiation (λ = 0.71073 Å) on a Rigaku XtaLAB mini diffractometer (Tokyo, Japan).

For structure **1** the intensity data were corrected for absorption [50] and decay using CrystalClear [51]. Final cell constants were calculated from the xyz centroids of strong reflections from the actual data collection after integration using CrystalClear. The structure of 1 was solved and refined using SHELXL-2013 [52] within the CrystalStructure program suite [53].

For structure **2** the intensity data were corrected for absorption and decay using spherical harmonics, implemented in SCALE3 ABSPACK scaling algorithm [54]. Final cell constants were calculated from the xyz centroids of strong reflections from the actual data collection after integration using CrysAlisPro v.171.40.80a [55]. The structure of **2** was solved using Olex2.solve v.1.3 [56] and refined using SHELXL v. 2018/3 [52] within the Olex2 v.1.3 program.

For both structures **1** and **2**, all non-hydrogen atoms were refined with anisotropic displacement parameters. All of the hydrogen atoms in each structure were placed in ideal positions (except H1 in structure **1**) and refined as riding atoms with relative isotropic displacement parameters. Attempts to refine the positions of hydrogens H1 and H2 of structure 2 resulted in significantly different N-H bond lengths (0.894 Å and 0.715 Å). As there does not appear to be a chemical reason for this large difference, both hydrogens were placed geometrically.

### 3.2. Computational Studies

The interaction energy and the electronic structures of the dimers studied here were evaluated with MP2 calculations and compared with Density Functional Theory (DFT) calculations with the ωB97X-D functional. 

The MP2 method presents a high electronic correlation in a dimer with a reasonable CPU time. This method has presented good results in recent studies of non-covalent interactions with halogen atoms [18,23,38,57].

The functional ωB97X-D [58] is a long-range-corrected hybrid functional that includes Grimme’s empirical dispersion model D2 [59], which is crucial for the dimers studied here. This functional shows good performance in treating non-covalent interactions [46] and for calculations of electronic properties [58]. The ωB97X-D was recently also employed with reasonable accuracy for descriptions of halogen interactions [20,60]. 

Both, MP2 and ωB97X-D functional were coupled with the df2-TZVP [61] basis set. These MP2/df2-TZVP and ωB97X-D/df2-TZVP theoretical levels methods have been shown to be a prominent combination for a good description of the interaction energy and molecular orbital in halogenic complexes [18,23]. These calculations were performed with the quantum chemistry Gaussian 16 [62] suite of programs. 

The interaction energies $E_{int}$, were calculated utilizing the supramolecular approach, in which the energy of the dimer ($E_{dimer}$) is computed and then subtracts the energies of the monomers ($E_{mon1}+E_{mon2}$),

(1) $$E_{int}=E_{dimer}-\left(E_{mon1}+E_{mon2}\right)$$

The Basis Set Superposition Error (BSSE) effects were corrected using the counterpoise method [63]. 

To analyze the orbital bonding mechanism of stabilizing charge–transfer interactions, the Natural Bond Orbital (NBO) theory was employed. NBO analyses allow us to understand the electron density delocalization from occupied NBOs (donor orbitals) and unoccupied NBOs, (acceptor orbitals) [64].

The intermolecular interaction was investigated employing the Quantum Theory of Atom and Molecules (QTAIM) [33,34,65,66,67] and Reduced Density Gradient (RDG) [39,68]. QTAIM and RDG analyses are based on the analysis of the electron density (ρ) and the second eigenvalue $\left(\lambda_{2}\right)$ of the Hessian matrix $\left(\nabla^{2}\rho =\lambda_{1}+\lambda_{2}+\lambda_{3}\right)$ at the called Bond Critical Points (BCPs) in the intermolecular region of interacting molecules. Currently, QTAIM and RDG approaches have found large applicability for the study of many electronic properties, particularly in the study of weakly interacting systems [69,70,71,72]. Recently, the combination of these two methods was found to be useful on description of the intermolecular halogen interactions [37,73]. 

Taking advantage of the QTAIM parameters, the contact energy, $E_{cont}$, for each pair of atom interactions was also estimated. For the determination of the $E_{cont}$, a set of equations used for estimation of the energy of each atom pair contact was employed. For the halogen type interaction energy, $E_{XB}$, the Tsirelson et al. [42], Bauzá et al. [18] and Kuznetsov [41] procedures were employed. These procedures correlate the $E_{XB}$ energy with the potential energy density, $V_{BCP}$, and with the Lagrangian Kinect energy, $G_{BCP}$, at the BCP as presented by Equations (2)–(5). For the hydrogen bonds energy, $E_{HB}$, the Espinosa et Al.’s [43] procedure was employed according to Equation (6).


(2) $$E_{XB}^{a}\approx 0.58\left(V_{BCP}\right),\;$$

(3) $$E_{XB}^{b}\approx 0.57\left(-G_{BCP}\right),\;$$

(4) $$E_{XB}^{c}\approx 0.375\left(V_{BCP}\right),\;$$

(5) $$-E_{XB}^{d}\approx 0.128{\left(G_{BCP}\right)}^{2}-0.824\left(G_{BCP}\right)+1.66,\;$$

(6) $$E_{HB}^{}\approx 0.5\left(V_{BCP}\right),\;$$

As some complexes present more than one interaction and, sometimes with different types, the attainment of the QTAIM-based contact energies was conducted by the sum of the different atom pair energies in a complex. To this end, the following set of equations was employed.


(7) $$E_{cont}^{a}=\sum E_{HB}+\sum E_{XB}^{a},\;$$

(8) $$E_{cont}^{b}=\sum E_{HB}+\sum E_{XB}^{b},\;$$

(9) $$E_{cont}^{c}=\sum E_{HB}+\sum E_{XB}^{c},\;$$

(10) $$E_{cont}^{d}=\sum E_{HB}+\sum E_{XB}^{d},\;$$

(11) $$E_{cont}^{HB}=\sum E_{HB},\;$$

The use of the topological analysis proven by QTAIM in association with the methods presented by Equations (2)–(11) has presented sufficient accuracy to estimate the strength of the interaction in different complexes [18,20,73,74]. 

QTAIM and RDG properties were computed using the wavefunction analysis free program Multiwfn [75]. The wavefunction was obtained employing the Gaussian 16 suite of the programs [62]. The drawings of the isosurfaces and molecules for both QTAIM and RDG analysis were constructed with VMD software version 1.9.3 [76]. The RDG scatter plots were drawn using the free software Gnuplot version 5.2.8 [77]. For the MEP analysis, the Gaussian 16 suite of programs was again employed.

## 4. Conclusions

X-ray structural determinations and computational studies were used to investigate halogen interactions in two halogenated oxindoles. Comparative analyses of the interaction energies of supramolecular dimers engaged in a variety of interactions (Br···Br, C-H···Br, C-H···F, C-H···O, NH···O) were examined. Analysis of intermolecular features identified from the experimental structural data and their comparison to vdW contact differences suggested weak but attractive Type I Br···Br interactions in **1** but not **2**, with strong hydrogen-bonding interactions in both **1** and **2**. Computation investigations, employing QTAIM, RDG and NBO methods, were carried out at two levels of theory (MP2/def2-TZVP and ωB97X-D/def2-TZVP). These analyses confirmed the attractive but weak nature of the Br···Br interactions of 1. The BSSE interaction energy together with QTAIM results suggest that C-H···Br, C-H···F, and C-H···O interactions in these structures are relatively weak, while the observed N-H···O hydrogen bonding interactions are the strongest interactions investigated. Substitution of a hydrogen atom in **1** with a fluorine atom in **2** allowed for new intermolecular interaction types (C-H···F) and altered interactions involving bromine to include C-H···Br interactions but also exclude Type I contacts. MEP analysis provided evidence that inductive effects of the fluorine substitution also enhanced intermolecular interactions found in **2** relative to **1**. All intermolecular interactions studied, with the exception of the Type I halogen contacts of 1, involved pairwise and likely cooperative effects that are not captured in our analysis. However, detailed examination of competing intermolecular interaction strengths and the effects caused by small structural changes provide guidance in using halogens as structure directing features of crystal design. 

## Figures and Tables

**Table 1:** X-ray Crystal data and refinement parameters for Structures **1** and **2**.

Sample	1	2
Formula	C_8_H_6_BrNO	C_8_H_5_BrFNO
Formula mass (g mol^−1^)	212.05	230.04
Crystal System	Monoclinic	Monoclinic
Space Group	P 2_1_/c	P 2_1_/c
*a* (Å)	4.286(2)	7.4802(10)
*b* (Å)	12.704(6)	14.5825(16)
*c* (Å)	14.187(7)	14.0709(16)
β (°)	93.458(7)	95.616(11)
Z	4	8
*V* (Å^3^)	771.2(7)	1527.5(3)
calcd (g cm^−3^)	1.826	2.001
T (K)	173	173
μ (mm^−1^)	5.279	5.341
*F* (000)	416	896
Total Reflections	6480	13,980
Independent Reflections	1406	2791
Data/restraints/parameters	1406/0/104	2791/0/217
*R*_int_	0.0857	0.0599
R_1_ (*I* ≥ 2σ(*I*))	0.0455	0.0404
wR_2_ (F^2^) (*I* ≥ 2σ(*I*))	0.0978	0.1038
R_1_ (all data)	0.0635	0.0499
wR_2_ (F^2^) (all data)	0.1057	0.1123
GOF	1.038	1.081
CCDC deposition number	2101968	2101969

**Table 2:** Short Intermolecular Contacts in Structures **1** and **2**.

Short Intermolecular Contact Type	Structure 1 Contact Atoms	Structure 1 Distance (Å) (Distance—vdW (Å))	Structure 2 Contact Atoms	Structure 2 Distance (Å) (Distance—vdW(Å))
Br···Br	Br1···Br1^1^	3.525 −0.175)	NA	None < vdW
N-H···O	H1···O1^2^	2.047 (−0.673)	H1···O1^3^	1.917 (−0.803)
			H2···O2^4^	1.929 (−0.791)
C-H···F	NA	NA	H2A^5^···F2	2.555 (−0.115)
			H10B···F1^6^	2.646 (−0.024)
C-H···Br	None < vdW	NA	H2A···Br2	2.997 (−0.053) 2.979 (−0.071) 3.006 (−0.044) 2.917 (−0.133)
			H2B···Br1^7^	
			H15···Br1^8^	
			H10A···Br2^9^	
C-H···O	H7^10^···O1 H2B^11^···O1	2.492 (−0.228) 2.691 (−0.029)	H5^6^···O2	2.419 (−0.301) 2.498 (−0.222) 2.690 (−0.030)
			H13···O1^5^	
			H2B^3^···O2	

NA = not applicable; symmetry codes (1) 2 − x, −y, 1 − z (2) −x, −y, 2 − z (3) 1 − x, 1 − y, 1 − z (4) −x, 1 − y, 1 − z (5) 1 − x, 0.5 + y, 1.5 − z (6) −1 − x, 1.5 − y, −0.5 + z (7) 2 − x, −0.5 + y, 1.5 − z (8) 1 − x, −0.5 + y, 1.5 − z (9) x, 1.5 − y, −0.5 + z (10) −1 + x, 0.5 − y, 0.5 + z (11) – 1 + x,y,z.

**Table 3:** Values of the electron density $\rho_{BCP\;}$, Laplacian of the electron density, ∇^2^ρ_*BCP*_, energy density, $H_{BCP}$, Lagrangian kinetic energy, $G_{BCP}$, potential energy density, $V_{BCP}$ the ratio of Lagrangian kinetic energy over by potential energy density, $\left|G_{BCP}/V_{BCP}\right|$ and second eigenvalue, $\lambda_{2}$, obtained at the critical points of the dimers with quantum theory of atoms in molecules (QTAIM) calculation. All values are in atomic units. The results were obtained with the MP2/def2-TZVP theory level.

Dimer	CP	$\rho_{BCP\;}\times 10^{-2}$	$\nabla^{2}\rho_{\mathrm{BCP}}\times 10^{-2}$	$H_{BCP}\times 10^{-3}$	$G_{BCP}\times 10^{-2}$	$V_{BCP}\times 10^{-2}$	$\left|G_{BCP}/V_{BCP}\right|$	$\lambda_{2}\times 10^{-2}$
1-BrBr	1	0.637	2.588	1.354	0.512	−0.376	1.360	−0.285
2-CHBrBr	1	0.376	1.241	0.744	0.236	−0.162	1.460	−0.193
	2	0.282	1.098	0.709	0.204	−0.133	1.534	0.412
	3	0.481	1.642	0.837	0.327	−0.243	1.344	−0.333
1-CHOCHBr	1	0.285	0.980	0.592	0.186	−0.127	1.467	−0.182
	2	0.124	0.471	0.327	0.085	−0.052	1.623	0.432
	3	0.738	0.323	1.856	0.622	−0.437	1.425	−0.610
2a-CHOCHF	1	0.777	3.439	1.916	0.668	−0.477	1.402	−0.719
	2	0.158	0.778	0.510	0.143	−0.925	1.551	0.652
	3	0.595	2.909	1.701	0.557	−0.387	1.440	−0.551
2b-CHOCHF	1	0.898	3.970	2.248	0.768	−0.543	1.414	−0.893
	2	0.136	0.719	0.510	0.129	−0.776	1.657	0.306
	3	0.459	2.154	1.283	0.410	−0.282	1.455	−0.413
1-NHONHO	1	1.693	11.505	4.763	2.400	−1.924	1.248	−2.125
	2	0.265	1.612	0.875	0.315	−0.228	1.384	0.579
	3	1.693	11.505	4.763	2.400	−1.924	1.248	−2.125
2a-NHONHO	1	2.588	12.721	3.898	2.790	−2.401	1.162	−3.626
	2	0.392	2.332	1.398	0.443	−0.303	1.461	0.918
	3	2.588	12.721	3.898	2.790	−2.401	1.162	−3.626
2b-NHONHO	1	2.588	12.721	3.898	2.790	−2.401	1.162	−3.626
	2	0.392	2.332	1.398	0.443	−0.303	1.461	0.918
	3	2.588	12.721	3.898	2.790	−2.401	1.162	−3.626

**Table 4:** NBO donors and acceptors and their second-order perturbation energy *E*^(2)^ for dimers of **1** and **2**. LP, BD* stand for lone pair and anti-bonding orbital, respectively. The results were obtained with the MP2/def2-TZVP theory level.

Complex	Donor	Acceptor	*E*^(2)^ (kcal/mol)
1-BrBr	LP (1) Br	BD*(1) Br-C	0.79
2-CHBrBr	LP (2) Br	BD*(1) Br-C	0.27
	LP (2) Br	BD*(1) C-H	0.26
1-CHOCHBr	LP (1) O	BD*(1) C-H	0.37
2a-CHOCHF	LP (1) O	BD*(1) C-H	0.25
2b-CHOCHF	LP (1) O	BD*(1) C-H	0.65
1-NHONHO	LP (2) O	BD*(1) N-H	3.07
	LP (2) O	BD*(1) N-H	3.07
2a-NHONHO	LP (1) O	BD*(1) N-H	2.70
	LP (2) O	BD*(1) N-H	5.97
	LP (1) O	BD*(1) N-H	2.70
	LP (2) O	BD*(1) N-H	5.97
2b-NHONHO	LP (1) O	BD*(1) N-H	2.68
	LP (2) O	BD*(1) N-H	5.54
	LP (1) O	BD*(1) N-H	2.68
	LP (2) O	BD*(1) N-H	5.54

**Table 5:** Basis set superposition error (BSSE) estimated by the counterpoise method, interaction energy, $E_{int}$, interaction energy with BSSE correction, $E_{int}\left(BSSE\right)$, interaction hydrogen bond energy, $E_{cont}^{HB}$, and the interaction contact energy, $\;E_{cont}^{a,b,c,d}$, in kcal/mol. The results were obtained with the MP2/def2-TZVP theory level.

Complex	$E_{int}\left(BSSE\right)$	$E_{cont}^{HB}$	$E_{cont}^{a}$	$E_{cont}^{b}$	$E_{cont}^{c}$	$E_{cont}^{d}$
1-BrBr	−0.765	—	−1.370	−1.830	−0.885	−1.656
2-CHBrBr	−1.478	—	−1.473	−2.013	−0.952	−3.315
1-CHOCHBr	−3.219	−1.768	−2.051	−2.891	−1.326	−3.029
2a-CHOCHF	−2.958	−2.709	−3.143	−4.383	−2.032	−3.151
2b-CHOCHF	−2.999	−2.587	−3.001	−4.212	−1.940	−3.359
1-NHONHO	−10.942	−12.072	—	—	−9.054	—
2a-NHONHO	−11.465	−15.064	—	—	−11.298	—
2b-NHONHO	−11.782	−15.064	—	—	−11.298	—

$E_{cont}^{HB}=\sum E_{HB}$; $E_{cont}^{a}=\sum E_{HB}+\sum E_{XB}^{a}$;$\;E_{cont}^{b}=\sum E_{HB}+\sum E_{XB}^{b}$;$\;E_{cont}^{c}=\sum E_{HB}+\sum E_{XB}^{c}$; $E_{cont}^{d}=\sum E_{HB}+\sum E_{XB}^{d}$. Were: $E_{HB}\approx 0.5\left(V_{BCP}\right),$ [43];$\;E_{XB}^{a}\approx 0.58\left(V_{BCP}\right)$ [42];$\;E_{XB}^{b}\approx 0.57\left(-G_{BCP}\right)$ [42]; $E_{XB}^{c}\approx 0.375\left(V_{BCP}\right)$ [18]; $-E_{XB}^{d}\approx 0.128{\left(G_{BCP}\right)}^{2}-0.824(G_{BCP})+1.66$ [41].

## Data Availability

The data presented in this study are available in the article and in the Supplementary Material. CIFs are openly available in www.ccdc.cam.ac.uk/data_request/cif (accessed on 5 September 2021).

## Supplementary Materials

The following are available online. Supplementary figures and tables including calculation results at the ωB97X-D/df2-TZVP theoretical level and cartesian coordinates used for all supramolecular dimer calculations are available online. Figure S1: Presents a comparison of bond lengths observed in the X-ray structures of **1** and **2**. Figure S2: Displays the unit cells, packing diagrams, and π-stacking observed in the X-ray structures of **1** and **2**. Figure S3: Shows details of the C-H···F and C-H···O paired interactions in structure **2** with the R 2,2(8) motif for each unique molecule of **2**. Figures S4–S6: Presents the critical points, bond paths and isosurfaces for the dimers 1-BrBr, 2-CHBrBr, 1-CHOCHBr, 2a-CHOCHF,2b-CHOCHF, 1-NHONHO, 2a-NHONHO and 2b- NHONHO dimers obtained with ωB97XD/def2-TZVP theory level. Figure S7: Presents the scatter graph for the non-covalent interactions obtained under RDG scheme obtained with ωB97XD/def2-TZVP theory level. Figure S8: Shows the NBO orbital for the dimers for ωB97XD/def2-TZVP theory level. QTAIM and NBO parameters, obtained with ωB97XD/def2-TZVP theory level, are presented in Tables S1 and S2: respectively. Table S3: Presents the $E_{int}$ and $E_{cont}$ energy obtained with ωB97XD/def2-TZVP theory level. Table S4: Presents the cartesian coordinates of the supramolecular dimers used for the theoretical calculations.

## Sample Availability

Samples of the compounds are not available from the authors.
