# Supplementary material for: Halogen Interactions in Halogenated Oxindoles: Crystallographic and Computational Investigations of Intermolecular Interactions
Source: Molecules. 2021 Sep 9;26(18):5487. doi: 10.3390/molecules26185487 (PMC8464904; doi:10.3390/molecules26185487)
Supplement: Supplementary file 1 [file molecules-26-05487-s001.zip › molecules-1370634-supplementary.pdf]

Supplementary Information

Halogen interactions in Halogenated Oxindoles: Crystallographic and Computational Investigations of Intermolecular Interactions

Rodrigo A. Lemos Silva [1], Demetrio A. da Silva Filho [1,2], Megan E. Moberg [3], Ted M. Pappenfus [4], and Daron E. Janzen [3,*]

1 Institute of Physics, University of Brasilia, Brasilia, 70910-900, Brasilia, Brazil; silvarodrigo021@gmail.com
2 International Center for Condensed Matter Physics, Universidade de Brasilia, CP 04455, 70919-970 - Brasilia, Brazil; dasf.all@gmail.com
3 Department of Chemistry & Biochemistry, St. Catherine University, St. Paul, Minnesota, 55105, USA
4 Division of Science and Mathematics, University of Minnesota, Morris, Minnesota 56267, USA; pappe001@morris.umn.edu
* Correspondence: dejanzen@stkate.edu

**Table of Contents**	**Page**
**Figure S1.** Comparison of bond lengths observed in the X-ray structures of **1** and **2**. All bond lengths in units of Å. Hydrogens omitted for clarity. Structure **1** (left) and structure **2** (right).	3
**Figure S2.** Unit cells, packing diagrams, and π-stacking observed in the X-ray structures of **1** and **2**. Hydrogens omitted for clarity. Structure **1** (left) and structure **2** (right).	4
**Figure S3**. C-H…F and C-H…O paired interactions in structure **2** showing the R 2,2(8) motif for each unique molecule of **2**.	5
**Figure S4.** Critical points and RDG isosurfaces for 1-BrBr and 2-CHBrBr complexes obtained with ωB97XD/def2-TZVP theory level for. The orange points indicate bond critical points (BCP), yellow points indicate ring critical points (RCP) and yellow lines indicate the bond paths. The RDG isosurfaces where obtained with isovalues of 0.65 a.u. The green-colored region indicates a van der Waals interaction in Br…Br.	6
**Figure S5.** Critical points and RDG isosurfaces for 1-CHOCHBr, 2a-CHOCHF and 2b-CHOCHF complexes obtained with ωB97XD/def2-TZVP theory level for. The orange points indicate bond critical points (BCP), yellow points indicate ring critical points (RCP) and yellow lines indicate the bond paths. The RDG isosurfaces where obtained with isovalues of 0.65 a.u. The green-colored region indicates a van der Waals interaction in Br…H, O…H and in the F…H pairwise.	7
**Figure S6.** Critical points and RDG isosurfaces for 1-NHONHO, 2a-NHONHO and 2b-NHONHO complexes obtained with ωB97XD/def2-TZVP theory level for. The orange points indicate bond critical points (BCP), yellow points indicate ring critical points (RCP) and yellow lines indicate the bond paths. The RDG isosurfaces where obtained with isovalues of 0.65 a.u . The green blue-colored region indicates a possible hydrogen bound interaction between NH…O.	8
**Figure S7.** Scatter graph of RDG. Points in the blue region, where $sign(\lambda_2)\rho$ assumes negative values are indicative of strong attractive interactions such as a hydrogen bond. Points in the green region, for values of $sign(\lambda_2)\rho$ close to zero, are indicative of van der Waals contacts. Points in the red region, where $sign(\lambda_2)\rho$ assumes positive values, are indicative of repulsive effects. These results were obtained with ωB97XD/def2-TZVP theory level.	9
**Figure S8.** NBO orbitals obtained with ωB97XD /def2-TZVP theory level. The NBO second-order energy perturbation $E^{(2)}$ is given in kcal/mol. The threshold of 0.10 kcal/mol were employed for the NBO orbitals printing	10-11
**Table S1**. Values of the electron density ρ_{BCP} , Laplacian of the electron density, $\nabla^2\rho_{BCP}$, energy density, H_{BCP}, Lagrangian kinetic energy, G_{BCP} , potential energy density, V_{BCP} , ratio of Lagrangian kinetic energy over by potential energy density, $\lvert G_{BCP}/V_{BCP}\rvert$ and second eigenvalue, λ_2, obtained at the critical points of the dimers 1 and 2 with quantum theory of	12

atoms in molecules (QTAIM) calculation. All values are in atomic units. The results were obtained with ωB97XD/def2-TZVP theory level.	
**Table S2.** NBO donors and acceptors and their second-order perturbation energy $E^{(2)}$ for dimer **1** and **2**. LP, BD* stand for lone pair and anti-bonding orbital, respectively. The results were obtained with ωB97XD/def2-TZVP theory level.	13
**Table S3.** Basis set superposition error (BSSE) estimated by the counterpoise method, interaction energy, E_{int}, interaction energy with BSSE correction, $E_{int}(BSSE)$, interaction hydrogen bond energy, E_{int}^{HB}, and the interaction contact energy, $E_{int}^{a,b,c,d}$, in kcal/mol. The results were obtained with ωB97XD/def2-TZVP theory level.	14
**Table S4.** Cartesian coordinates of the supramolecular dimers used for the theoretical calculations.	15-22
**References**	23

Figure S1. Comparison of bond lengths observed in the X-ray structures of **1** and **2**. All bond lengths in units of Å. Hydrogens omitted for clarity. Structure **1** (left) and structure **2** (right).

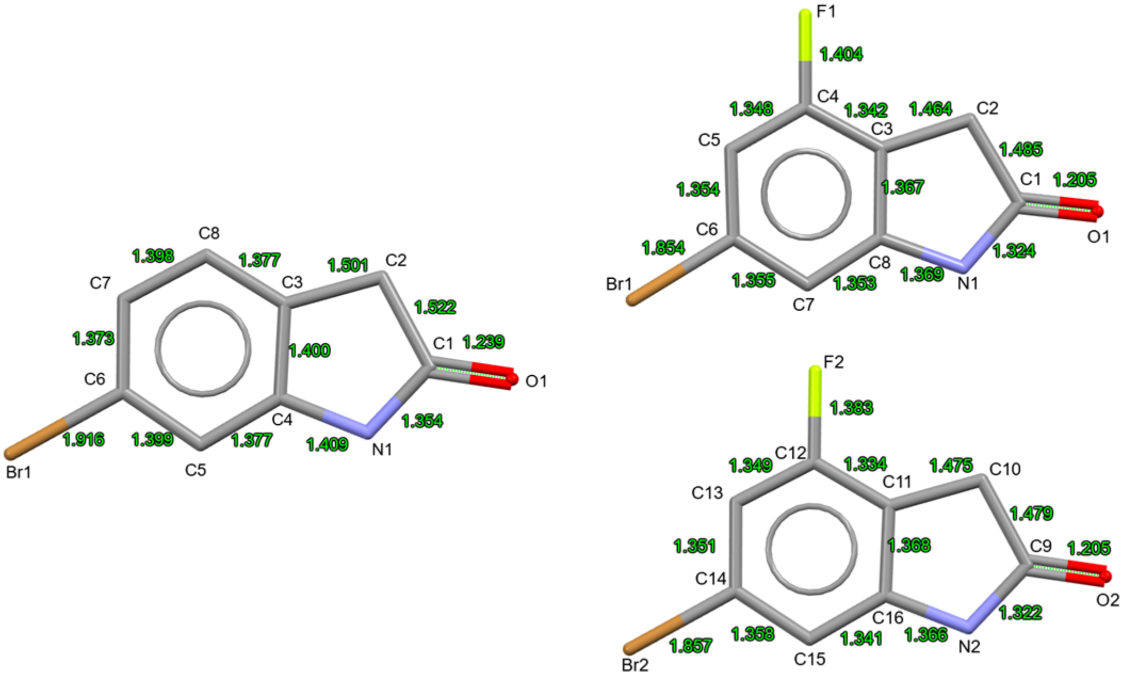

Figure S2. Unit cells, packing diagrams, and π-stacking observed in the X-ray structures of **1** and **2**. Hydrogens omitted for clarity. Structure **1** (left) and structure **2** (right).

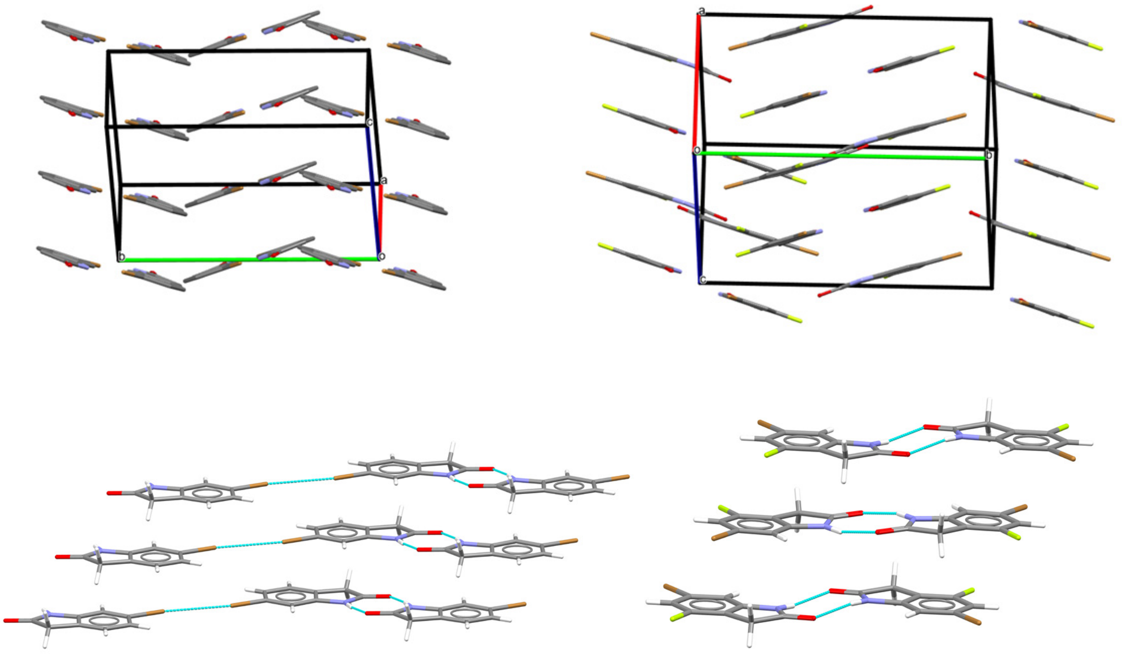

Figure S3. C-H…F and C-H…O paired interactions in structure **2** showing the R2,2(8) motif for each unique molecule of **2**.

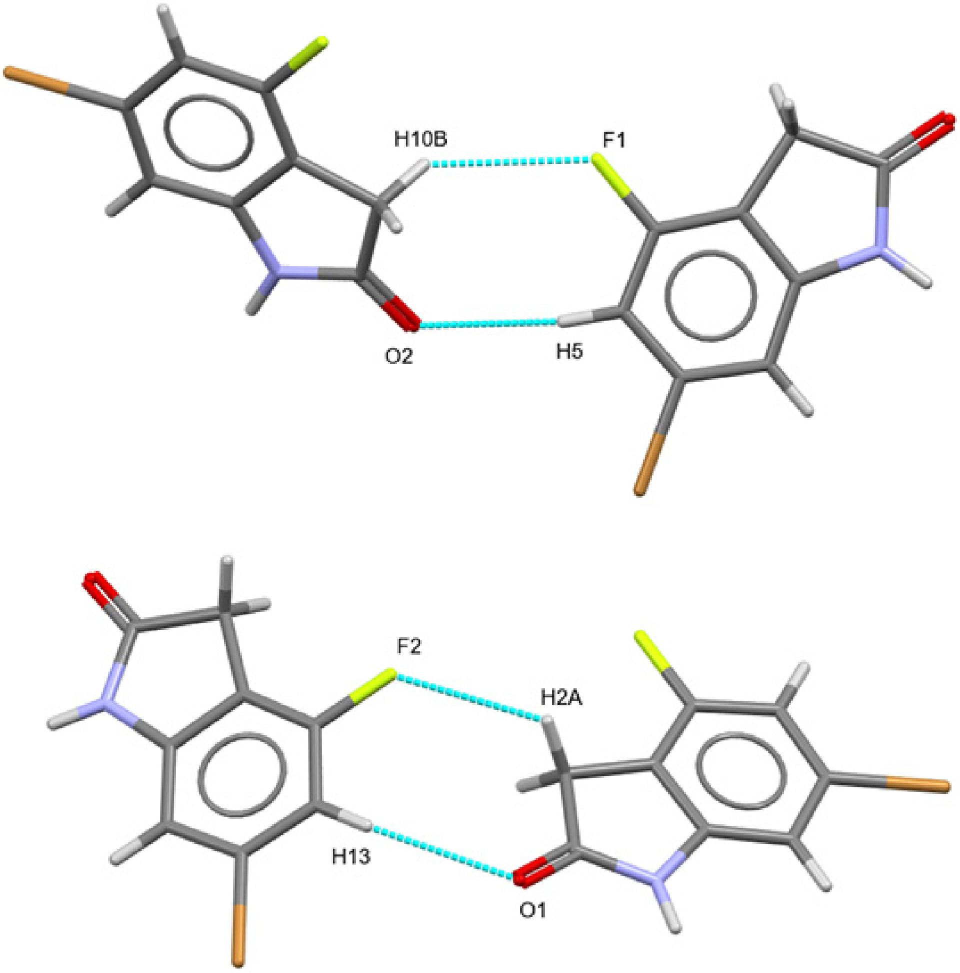

Figure S4. Critical points and RDG isosurfaces for 1-BrBr and 2-CHBrBr complexes obtained with ωB97XD/def2-TZVP theory level for. The orange points indicate bond critical points (BCP), yellow points indicate ring critical points (RCP) and yellow lines indicate the bond paths. The RDG isosurfaces where obtained with isovalues of 0.65 a.u. The green-colored region indicates a van der Waals interaction in Br…Br.

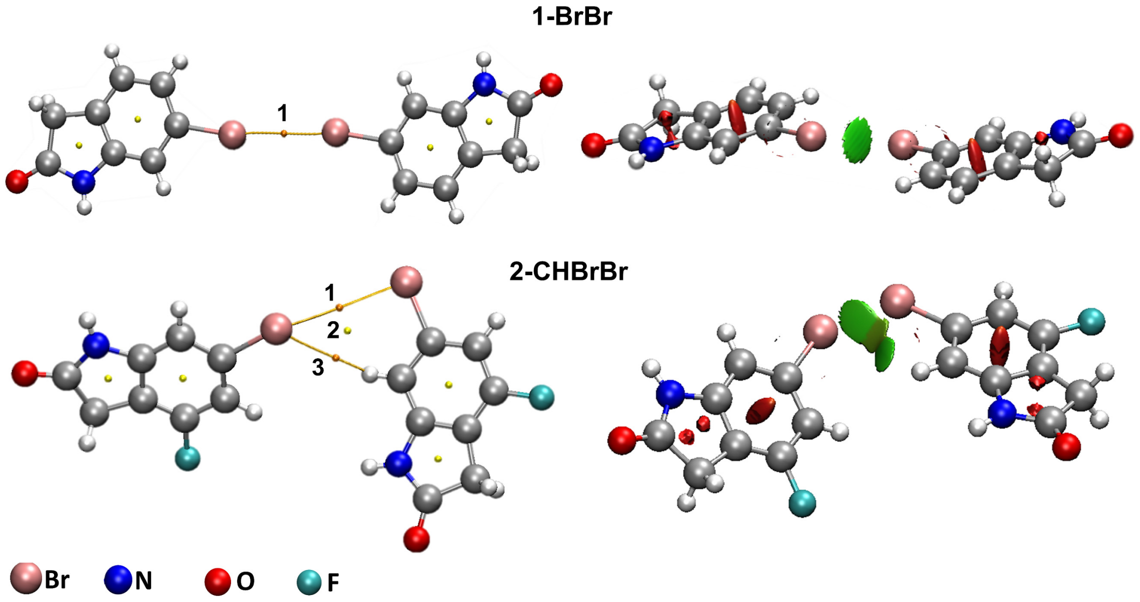

Figure S5. Critical points and RDG isosurfaces for 1-CHOCHBr, 2a-CHOCHF and 2b-CHOCHF complexes obtained with ωB97XD/def2-TZVP theory level for. The orange points indicate bond critical points (BCP), yellow points indicate ring critical points (RCP) and yellow lines indicate the bond paths. The RDG isosurfaces where obtained with isovalues of 0.65 a.u. The green-colored region indicates a van der Waals interaction in Br…H, O…H and in the F…H pairwise.

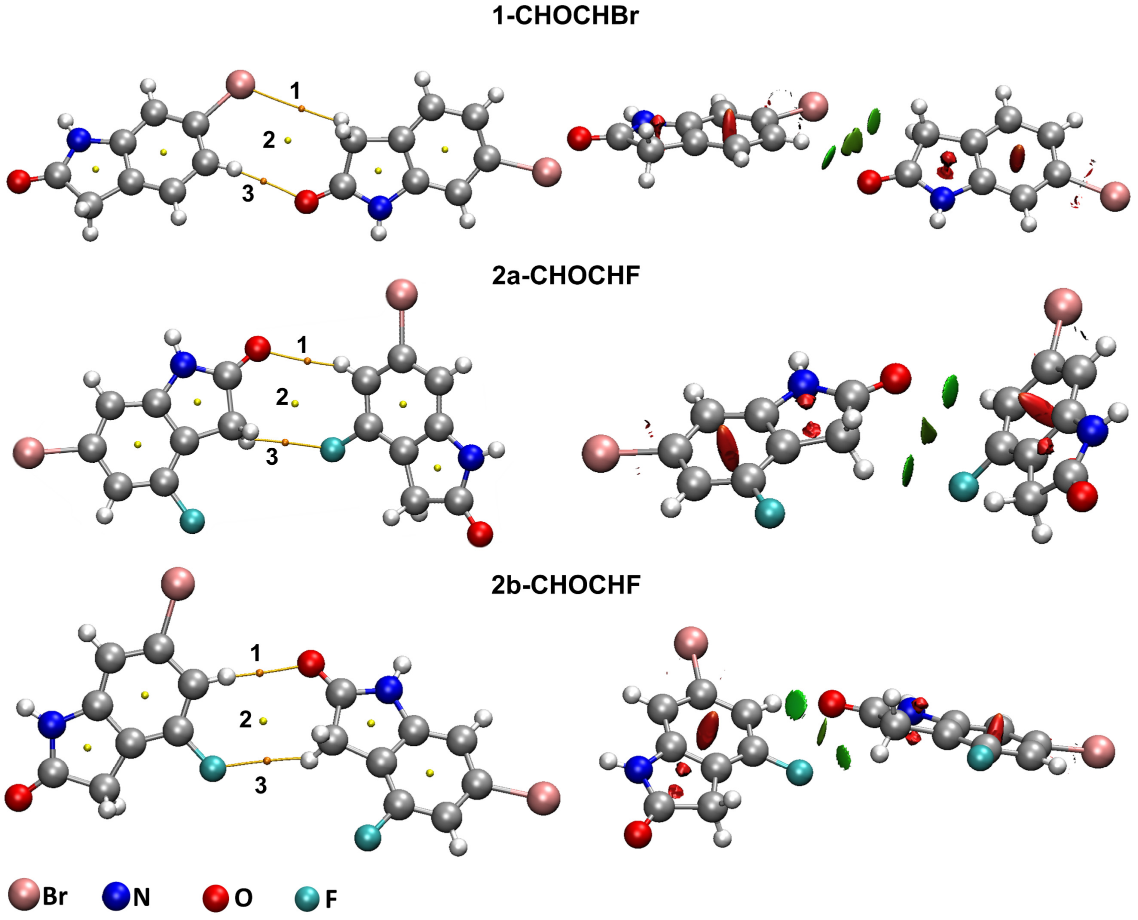

Figure S6. Critical points and RDG isosurfaces for 1-NHONHO, 2a-NHONHO and 2b-NHONHO dimers obtained with ωB97XD/def2-TZVP theory level. The orange points indicate bond critical points (BCP), yellow points indicate ring critical points (RCP) and yellow lines indicate the bond paths. The RDG isosurfaces where obtained with isovalues of 0.65 a.u . The green blue-colored region indicates a possible hydrogen bound interaction between NH…O.

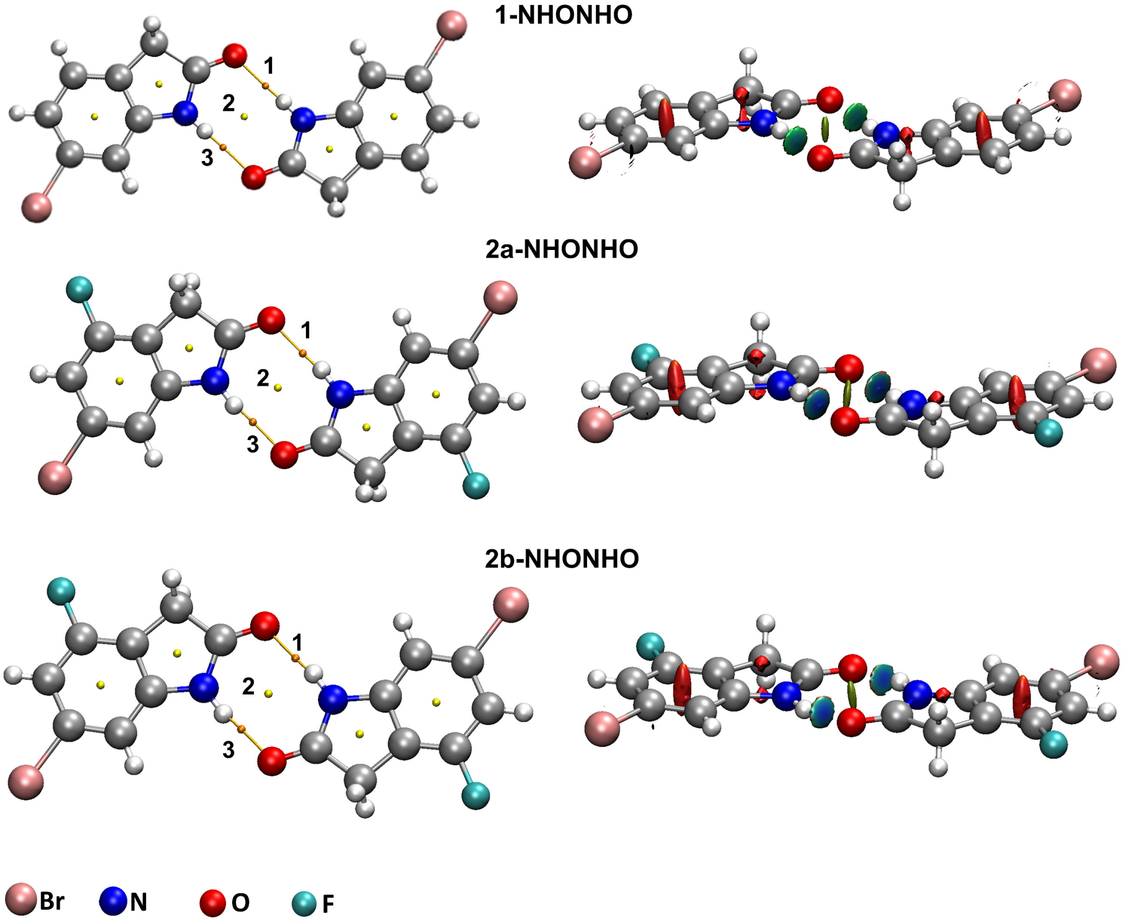

Figure S7. Scatter graph of RDG. Points in the blue region, where $sign(\lambda_2)\rho$ assumes negative values are indicative of strong attractive interactions like a hydrogen bond. Points in the green region, for values of $sign(\lambda_2)\rho$ close to zero, are indicative of van der Waals contacts. Points in the red region, where $sign(\lambda_2)\rho$ assumes positive values, are indicative of repulsive effects. These results were obtained with ωB97XD/def2-TZVP theory level.

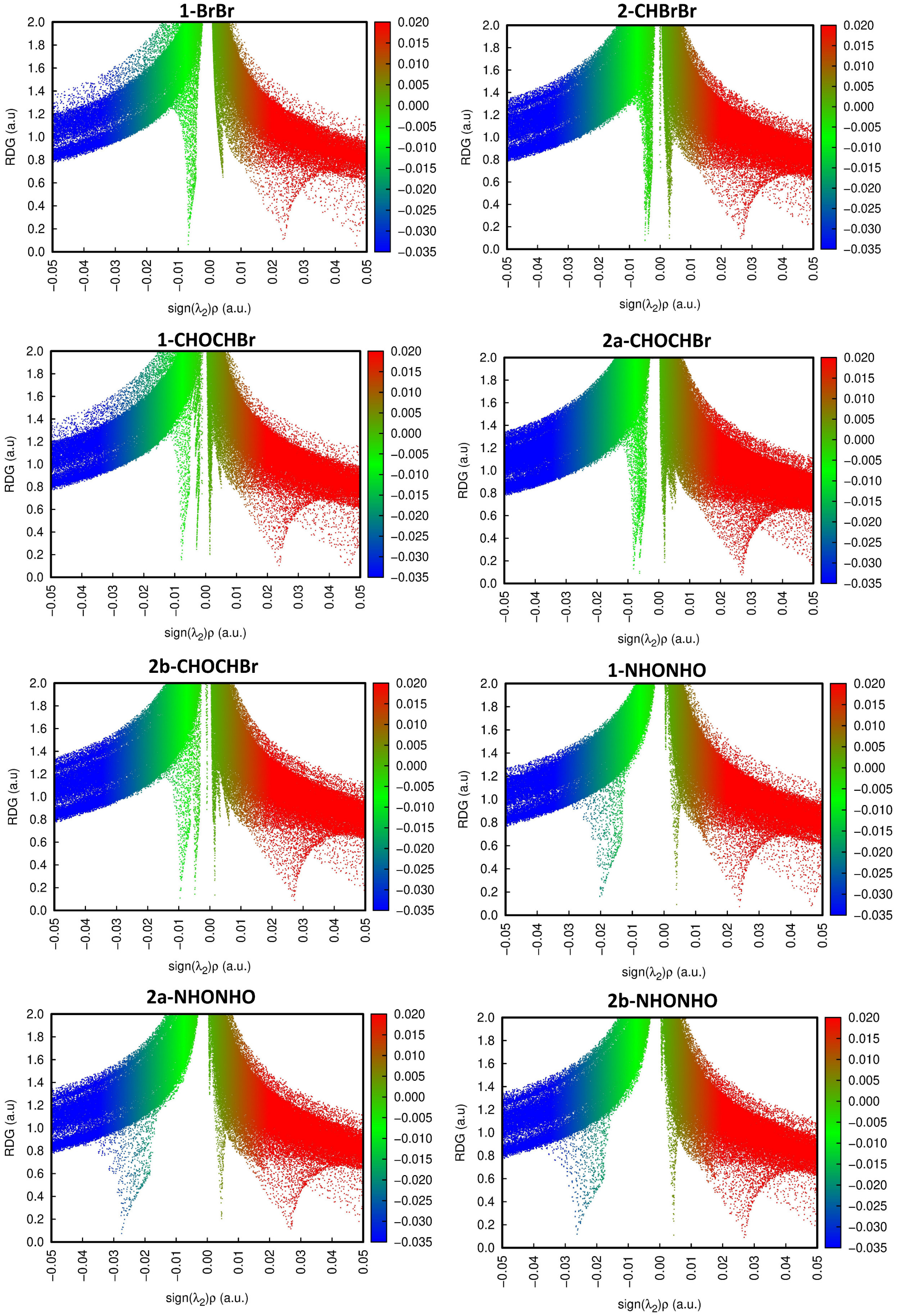

Figure S8. NBO orbitals obtained with ωB97XD /def2-TZVP theory level. The NBO second-order energy perturbation $E^{(2)}$ is given in kcal/mol. The threshold of 0.10 kcal/mol were employed for the NBO orbitals printing.

1-BrBr

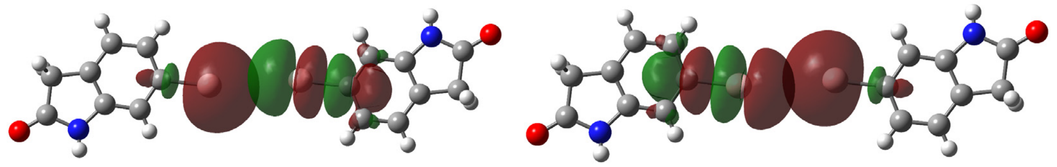

$E^{(2)} = 0.75$ $E^{(2)} = 0.75$

2-CHBrBr

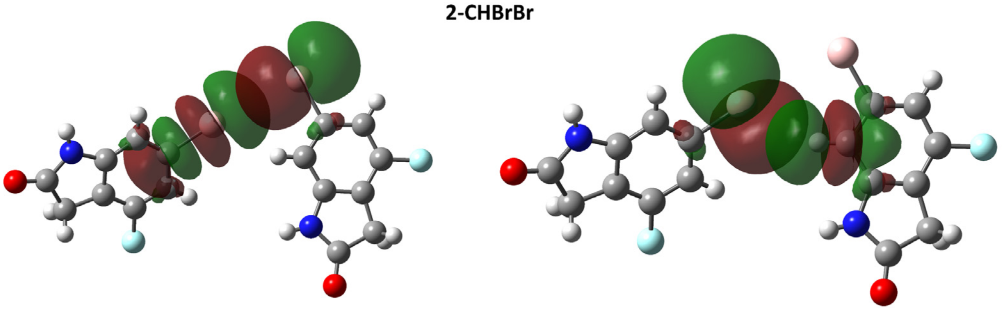

$E^{(2)} = 0.31$ $E^{(2)} = 0.36$

1-CHOCHBr

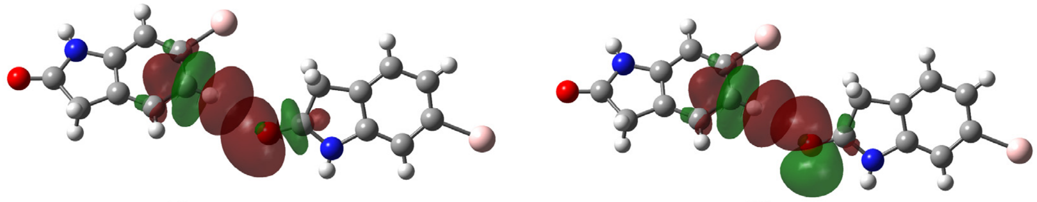

$E^{(2)} = 0.19$ $E^{(2)} = 0.53$

2a-CHOCHF

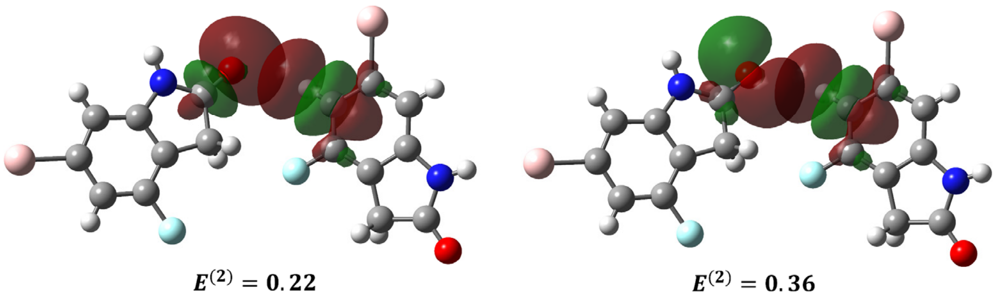

$E^{(2)} = 0.22$ $E^{(2)} = 0.36$

2b-CHOCHF

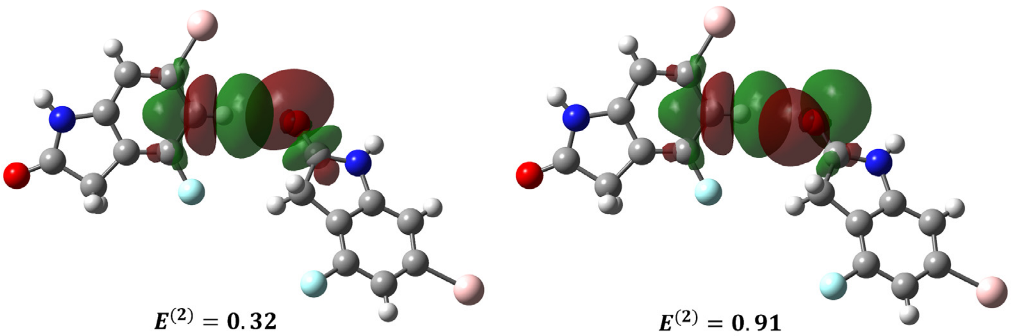

$E^{(2)} = 0.32$ $E^{(2)} = 0.91$

Figure S8. Continuing.

1-NHONHO

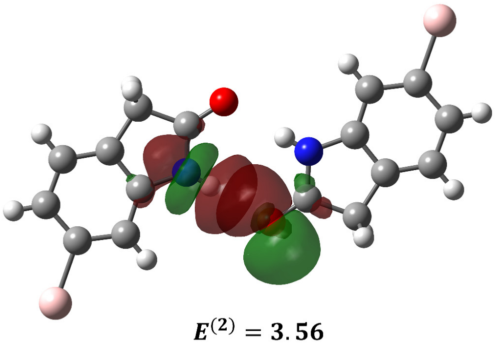

$E^{(2)} = 3.56$

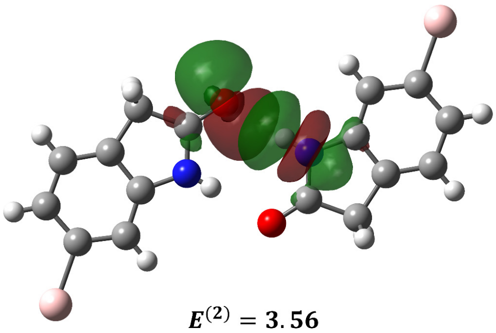

$E^{(2)} = 3.56$

2a-NHONHO

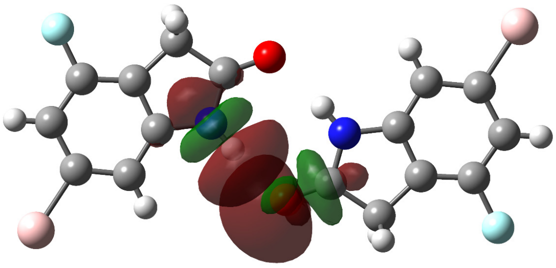

$E^{(2)} = 2.81$

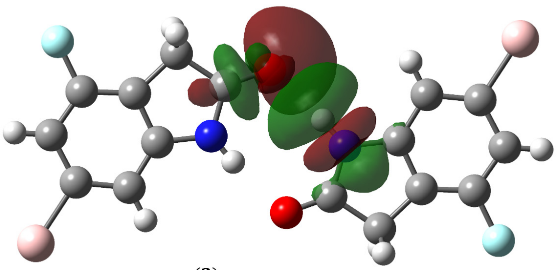

$E^{(2)} = 2.81$

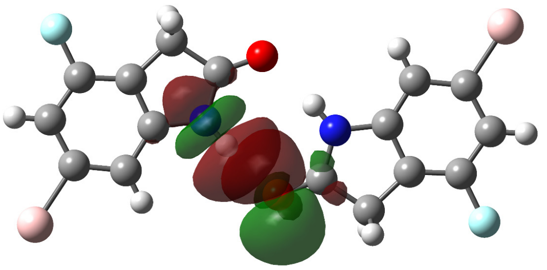

$E^{(2)} = 6.63$

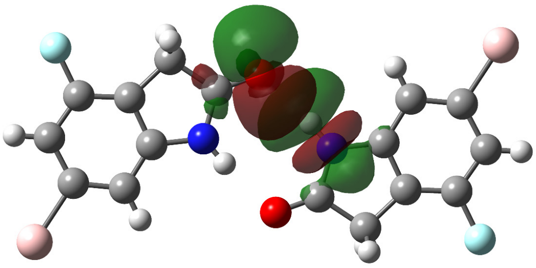

$E^{(2)} = 6.63$

2b-NHONHO

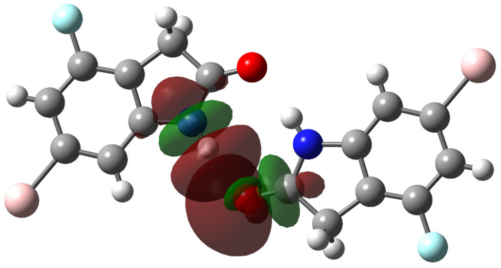

$E^{(2)} = 2.80$

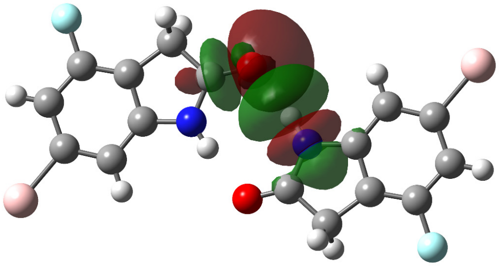

$E^{(2)} = 2.80$

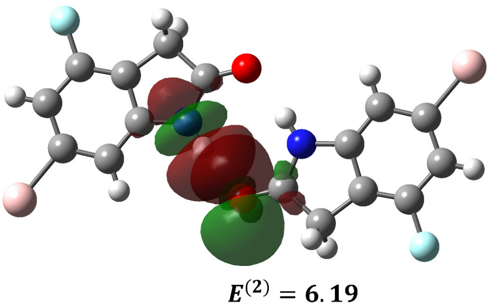

$E^{(2)} = 6.19$

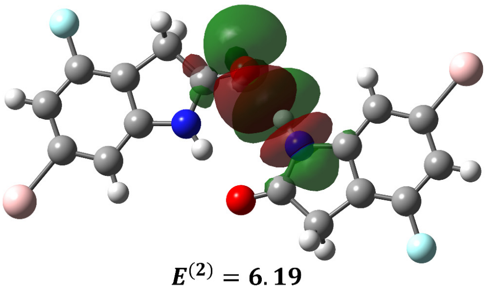

$E^{(2)} = 6.19$

QTAIM parameters

The QTAIM analysis was performed using the same theory level as used for interaction energy calculation, e.g. MP2/df2-TZVP and ωB97XD/df2-TZVP theoretical levels. All QTAIM analysis was performed using the free program Multiwfn [1]. The wave functions employed for the obtention of the topological QTAIM parameters were obtained from theoretical calculations with the quantum chemistry Gaussian 16 [2] suite of programs. The draw of the isosurfaces and molecules for both QTAIM were made with VMD software version 1.9.3 [3]. Table SY presents the QTAIM parameters results for ωB97XD/def2-TZVP theory level. The results for MP2/def2-TZVP are presented in Table 3 in the main text.

Table S1. Values of the electron density ρ_{BCP}, Laplacian of the electron density, $\nabla^2{}_{\rho BCP}$, energy density, H_{BCP}, Lagrangian kinetic energy, G_{BCP}, potential energy density, V_{BCP}, ratio of Lagrangian kinetic energy over by potential energy density, $|G_{BCP}/V_{BCP}|$ and second eigenvalue, λ_2, obtained at the critical points of the dimers 1 and 2 with quantum theory of atoms in molecules (QTAIM) calculation. All values are in atomic units. The results were obtained with ωB97XD/def2-TZVP theory level.

Complex	CP	$\rho_{BCP} \times 10^{-2}$	$\nabla^2\rho_{BCP} \times 10^{-2}$	$H_{BCP} \times 10^{-3}$	$G_{BCP} \times 10^{-2}$	$V_{BCP} \times 10^{-2}$	$\lvert G_{BCP}/V_{BCP}\rvert$	$\lambda_2 \times 10^{-2}$
1-BrBr	1	0.637	2.588	1.354	0.512	-0.376	1.360	-0.329
2-CHBrBr	1	0.389	1.175	0.695	0.224	-0.155	1.449	-0.191
	2	0.497	1.555	0.781	0.311	-0.233	1.336	-0.339
	3	0.304	1.052	0.664	0.197	-0.130	1.511	0.384
1-CHOCHBr	1	0.290	0.935	0.568	0.177	-0.120	1.473	-0.182
	2	0.788	3.078	1.699	0.599	-0.430	1.396	-0.744
	3	0.138	0.474	0.327	0.086	-0.053	1.616	0.106
2a-CHOCHF	1	0.822	3.262	1.769	0.639	-0.462	1.383	-0.748
	2	0.628	2.800	1.60	0.541	-0.381	1.418	-0.559
	3	0.188	0.814	0.512	0.152	-0.100	1.516	0.219
2b-CHOCHF	1	0.957	3.740	2.014	0.734	-0.532	1.378	-0.948
	2	0.487	2.090	1.212	0.401	-0.280	1.433	-0.426
	3	0.161	0.751	0.524	0.135	-0.083	1.633	0.332
1-NHONHO	1	2.018	9.124	3.809	1.900	-1.519	1.251	-2.558
	2	2.018	9.124	3.809	1.900	-1.519	1.251	-2.558
	3	0.398	1.864	1.024	0.364	-0.261	1.392	0.869
2a-NHONHO	1	2.746	11.603	2.721	2.629	-2.356	1.115	-3.831
	2	2.746	11.603	2.721	2.629	-2.356	1.115	-3.831
	3	0.456	2.363	1.330	0.458	-0.325	1.409	0.976
2b-NHONHO	1	2.657	11.308	2.908	2.536	-2.246	1.129	-3.676
	2	2.657	11.308	2.908	2.536	-2.246	1.129	-3.676
	3	0.445	2.235E	1.263	0.432	-0.306	1.413	1.067

NBO parameters

The NBO analysis was performed with MP2/df2-TZVP and ωB97XD/df2-TZVP theoretical levels. The NBO calculations and analysis were performed with the quantum chemistry Gaussian 16 [2] suite of programs. Table SZ presents the NBOs (donor orbitals) and unoccupied NBOs, (acceptor orbitals) calculated with ωB97XD/def2-TZVP theory level. The results for MP2/def2-TZVP are presented in Table 4 in the main text.

Table S2. NBO donors and acceptors and their second-order perturbation energy $E^{(2)}$ for dimer 1 and 2. LP, BD* stand for lone pair and anti-bonding orbital, respectively. The results were obtained with ωB97XD/def2-TZVP theory level.

Complex	Donor	Acceptor	$E^{(2)}$
1-BrBr	LP (2) Br	BD*(1) Br - C	0.75
2-CHBrBr	LP (2) Br	BD*(1) Br - C	0.31
	LP (2) Br	BD*(1) C - H	0.36
1-CHOCHBr	LP (1) O	BD*(1) C - H	0.19
	LP (2) O	BD*(1) C - H	0.53
2a-CHOCHF	LP (1) O	BD*(1) C - H	0.22
	LP (1) O	BD*(1) C - H	0.36
2b-CHOCHF	LP (1) O	BD*(1) C - H	0.32
	LP (1) O	BD*(1) C - H	0.91
1-NHONHO	LP (2) O	BD*(1) N - H	3.56
	LP (2) O	BD*(1) N - H	3.56
2a-NHONHO	LP (1) O	BD*(1) N - H	2.81
	LP (2) O	BD*(1) N - H	6.63
	LP (1) O	BD*(1) N - H	2.81
	LP (2) O	BD*(1) N - H	6.63
2b-NHONHO	LP (1) O	BD*(1) N - H	2.80
	LP (2) O	BD*(1) N - H	6.19
	LP (1) O	BD*(1) N - H	2.80
	LP (2) O	BD*(1) N - H	6.19

Interaction and contact energies

All theoretical investigations were done employing MP2/df2-TZVP and Density Functional Theory (DFT) ωB97XD/df2-TZVP theoretical levels. While MP2 method presents a high electronic correlation in a dimer with a reasonable CPU time, the ωB97XD functional shows good performance to treat non-covalent interactions[4]and for calculations of electronic properties[5]. Both, MP2 and ωB97XD functional were coupled with the df2-TZVP[6] basis set. These MP2/df2-TZVP and ωB97XD/df2-TZVP theoretical levels methods have been shown to be a prominent combination for a good description of the interaction energy and molecular orbital in halogenic complexes[7,8]. All these calculations were performed with the quantum chemistry Gaussian 16 [2] suite of programs. Table S2 presents the results for interaction energies obtained by mean of the supramolecular approach (Eq. (1) in the main text) and contact energies estimated by Tsirelson et. al. [9] and Bauzá et. al. procedures, as presented by the Equations 2, 3 and 4 in the main text, obtained with ωB97XD/def2-TZVP theory level. The results for MP2/def2-TZVP are presented in Table 5 in the main text.

Table S3. Basis set superposition error (BSSE) estimated by the counterpoise method, interaction energy, E_{int}, interaction energy with BSSE correction, $E_{int}(BSSE)$, interaction hydrogen bond energy, E_{cont}^{HB}, and the interaction contact energy, $E_{cont}^{a,b,c,d}$, in kcal/mol. The results were obtained with ωB97XD/def2-TZVP theory level.

Complex	$\mathbf{E_{int}(BSSE)}$	E_{cont}^{HB}	E_{cont}^{a}	E_{cont}^{b}	E_{cont}^{c}	E_{cont}^{d}
1-BrBr	0.035	---	-1.370	-1.830	-0.885	-1.656
2-CHBrBr	-1.354	---	-1.410	-1.913	-0.911	-3.316
1-CHOCHBr	-3.387	-1.724	-2.000	-2.777	-1.293	-3.006
2a-CHOCHF	-3.111	-2.644	-3.067	-4.217	-1.983	-3.104
2b-CHOCHF	-3.215	-2.549	-2.957	-4.059	-1.912	-3.327
1-NHONHO	-11.699	-9.533	---	---	-7.150	---
2a-NHONHO	-12.579	-14.787	---	---	-11.090	---
2b-NHONHO	-12.884	-14.091	---	---	-10.568	---

$E_{cont}^{HB} = \sum E_{HB}$; $E_{cont}^{a} = \sum E_{HB} + \sum E_{XB}^{a}$; $E_{cont}^{b} = \sum E_{HB} + \sum E_{XB}^{b}$; $E_{cont}^{c} = \sum E_{HB} + \sum E_{XB}^{c}$; $E_{cont}^{d} = \sum E_{HB} + \sum E_{XB}^{d}$.
Were: $E_{HB} \approx 0.5(V_{BCP})$,[10]; $E_{XB}^{a} \approx 0.58(V_{BCP})$[9]; $E_{XB}^{b} \approx 0.57(-G_{BCP})$[9]; $E_{XB}^{c} \approx 0.375(V_{BCP})$[7] ; $-E_{XB}^{d} \approx 0.128(G_{BCP})^2 - 0.824(G_{BCP}) + 1.66$[11].

Cartesian Coordinates

All cartesian coordinates were extracted direct from X-ray crystallography data of 1 or 2. Single point calculations were performed to obtain the electronic and energetic properties. To prevent the molecule reorientation, the NoSymm keyword was employed in all theoretical calculations inputs. For the self-constituent field calculation (SCF), to guarantee the energy conversion, the quadratically convergent (QC) with an extra step (XQC) was used. Additionally, the NoVarAcc option was also employed to prevent the use of modest integral accuracy in the early direct SCF.

Table S4. Cartesian coordinates of the supramolecular dimers used for the theoretical calculations.

**1-BrBr**			
**Atoms**	**x**	**y**	**z**
Br	3.13693400	0.52747000	8.59993700
O	-0.65812300	1.62992300	15.04765900
N	0.32865500	0.84989800	13.12315600
C	0.09208200	1.78491200	14.07337000
C	0.88847000	3.03371500	13.72500500
H	0.29399000	3.81755200	13.61171600
H	1.56170200	3.23570900	14.42173500
C	1.54494900	2.65640600	12.42925800
C	1.20263800	1.33265000	12.12904200
C	1.66695200	0.66187800	11.02022200
H	1.44029000	-0.24264600	10.84179100
C	2.49629000	1.40760300	10.17621600
C	2.86311200	2.70849300	10.41837200
H	3.43308700	3.17218900	9.81652300
C	2.37951400	3.33607000	11.56967500
H	2.62735100	4.23424300	11.75943500
H	-0.00547200	0.12704000	13.21237100
Br	4.57935100	-0.52747000	5.56123300
O	8.37440800	-1.62992300	-0.88648900
N	7.38763000	-0.84989800	1.03801400
C	7.62420300	-1.78491200	0.08779900
C	6.82781500	-3.03371500	0.43616400
H	7.42229500	-3.81755200	0.54945300
H	6.15458300	-3.23570900	-0.26056600
C	6.17133600	-2.65640600	1.73191100
C	6.51364700	-1.33265000	2.03212800
C	6.04933300	-0.66187800	3.14094700
H	6.27599500	0.24264600	3.31937800
C	5.21999400	-1.40760300	3.98495300
C	4.85317300	-2.70849300	3.74279700
H	4.28319800	-3.17218900	4.34464700
C	5.33677000	-3.33607000	2.59149400
H	5.08893400	-4.23424300	2.40173400
H	7.72175700	-0.12704000	0.94879800

**2-CHBrBr**			
**Atoms**	**x**	**y**	**z**

Br	5.78439100	13.13693700	8.66107900
F	5.85219200	9.29780200	12.09078200
N	4.02482800	8.27702700	8.02812700
H	3.77131900	8.33276100	7.20761400
O	3.31724900	6.18298000	8.43842500
C	3.87710600	7.19500600	8.77730700
C	4.95207000	8.85157700	9.96339200
C	4.62772000	9.29780200	8.71289100
C	5.77348800	11.01270400	10.44930800
H	6.15769300	11.61065900	11.05019200
C	4.87426200	10.55918800	8.28999000
H	4.66225800	10.83815100	7.42910500
C	4.54106100	7.45165800	10.08102000
H	3.92437300	7.32710800	10.81948700
H	5.30728900	6.86930500	10.20553800
C	5.52077900	9.73527700	10.79799200
C	5.44683400	11.39330700	9.19180600
Br	3.00693500	12.90595000	5.74361900
F	2.75945400	9.43998100	1.99295800
O	0.20014100	13.13037500	-1.07685800
N	0.97830000	13.52818500	0.99003800
H	0.74419400	14.35330700	1.04927200
C	1.39717400	11.45601200	0.16103900
H	2.13544300	11.29598400	-0.44701500
H	0.74637700	10.74284000	0.06646000
C	2.78996700	11.08124200	3.66047900
H	3.19462200	10.47926200	4.24243000
C	0.78069100	12.77864500	-0.08121900
C	1.59780600	12.83697500	1.99267800
C	1.92095500	13.25549300	3.22497400
H	1.74783600	14.12479900	3.50541900
C	2.45658100	10.72542900	2.40297700
C	2.51300900	12.34700300	4.04277000
C	1.86680800	11.56975500	1.55437300

**1-CHOCHBr**			
**Atoms**	**x**	**y**	**z**

Br	3.13693400	0.52747000	8.59993700
O	-0.65812300	1.62992300	15.04765900
N	0.32865500	0.84989800	13.12315600
C	0.09208200	1.78491200	14.07337000
C	0.88847000	3.03371500	13.72500500
H	0.29399000	3.81755200	13.61171600
H	1.56170200	3.23570900	14.42173500
C	1.54494900	2.65640600	12.42925800
C	1.20263800	1.33265000	12.12904200
C	1.66695200	0.66187800	11.02022200
H	1.44029000	-0.24264600	10.84179100
C	2.49629000	1.40760300	10.17621600
C	2.86311200	2.70849300	10.41837200
H	3.43308700	3.17218900	9.81652300
C	2.37951400	3.33607000	11.56967500
H	2.62735100	4.23424300	11.75943500
H	-0.00547200	0.12704000	13.21237100
Br	-1.57692400	5.82453000	15.68052100
O	-5.37198100	4.72207700	22.12824300
N	-4.38520300	5.50210200	20.20374000
C	-4.62177600	4.56708800	21.15395500
C	-3.82538800	3.31828500	20.80559000
H	-4.41986800	2.53444800	20.69230100
H	-3.15215600	3.11629100	21.50232000
C	-3.16890900	3.69559400	19.50984300
C	-3.51122000	5.01935000	19.20962600
C	-3.04690500	5.69012200	18.10080700
H	-3.27356800	6.59464600	17.92237600
C	-2.21756700	4.94439700	17.25680100
C	-1.85074600	3.64350700	17.49895700
H	-1.28077100	3.17981100	16.89710700
C	-2.33434300	3.01593000	18.65026000
H	-2.08650700	2.11775700	18.84002000
H	-4.71933000	6.22496000	20.29295600

	**2a-CHOCHF**		
**Atoms**	**x**	**y**	**z**

Br	5.78439100	13.13693700	8.66107900
F	5.85219200	9.29780200	12.09078200
N	4.02482800	8.27702700	8.02812700
H	3.77131900	8.33276100	7.20761400
O	3.31724900	6.18298000	8.43842500
C	3.87710600	7.19500600	8.77730700
C	4.95207000	8.85157700	9.96339200
C	4.62772000	9.29780200	8.71289100
C	5.77348800	11.01270400	10.44930800
H	6.15769300	11.61065900	11.05019200
C	4.87426200	10.55918800	8.28999000
H	4.66225800	10.83815100	7.42910500
C	4.54106100	7.45165800	10.08102000
H	3.92437300	7.32710800	10.81948700
H	5.30728900	6.86930500	10.20553800
C	5.52077900	9.73527700	10.79799200
C	5.44683400	11.39330700	9.19180600
Br	3.09627600	1.67655000	8.25974300
F	3.34375600	5.14251900	12.01040300
O	5.90306900	1.45212500	15.08022000
N	5.12491100	1.05431500	13.01332400
H	5.35901600	0.22919300	12.95408900
C	4.70603600	3.12648800	13.84232300
H	3.96776800	3.28651600	14.45037700
H	5.35683300	3.83966000	13.93690100
C	3.31324300	3.50125800	10.34288300
H	2.90858800	4.10323800	9.76093100
C	5.32251900	1.80385500	14.08458100
C	4.50540400	1.74552500	12.01068300
C	4.18225500	1.32700800	10.77838700
H	4.35537400	0.45770100	10.49794200
C	3.64663000	3.85707100	11.60038400
C	3.59020100	2.23549700	9.96059100
C	4.23640200	3.01274500	12.44898800

2b-CHOCHF

Atoms	x	y	z

Br	2.31844000	8.96780000	12.74529900
F	2.07095900	12.43376900	8.99463900
O	-0.48835400	8.74337500	5.92482200
N	0.28980500	8.34556500	7.99171800
H	0.05569900	7.52044300	8.05095200
C	0.70868000	10.41773800	7.16271900
H	1.44694800	10.57776600	6.55466500
H	0.05788200	11.13091000	7.06814100
C	2.10147200	10.79250800	10.66215900
H	2.50612700	11.39448800	11.24411100
C	0.09219600	9.09510500	6.92046100
C	0.90931100	9.03677500	8.99435900
C	1.23246000	8.61825700	10.22665500
H	1.05934100	7.74895100	10.50710000
C	1.76808600	11.14832100	9.40465700
C	1.82451400	9.52674700	11.04445100
C	1.17831300	10.30399400	8.55605400
Br	-1.00731400	8.73681300	1.65939800
F	-0.93951300	12.57594800	5.08910200
N	-2.76687700	13.59672300	1.02644600
H	-3.02038600	13.54098900	0.20593300
O	-3.47445600	15.69077000	1.43674500
C	-2.91460000	14.67874400	1.77562600
C	-1.83963500	13.02217200	2.96171100
C	-2.16398600	12.57594800	1.71121100
C	-1.01821700	10.86104600	3.44762800
H	-0.63401200	10.26309100	4.04851200
C	-1.91744300	11.31456200	1.28830900
H	-2.12944700	11.03559900	0.42742500
C	-2.25064400	14.42209200	3.07933900
H	-2.86733200	14.54664200	3.81780600
H	-1.48441600	15.00444500	3.20385700
C	-1.27092600	12.13847300	3.79631100
C	-1.34487100	10.48044300	2.19012600

1-NHONHO			
**Atoms**	**x**	**y**	**z**

Br	3.13693400	0.52747000	8.59993700
O	-0.65812300	1.62992300	15.04765900
N	0.32865500	0.84989800	13.12315600
C	0.09208200	1.78491200	14.07337000
C	0.88847000	3.03371500	13.72500500
H	0.29399000	3.81755200	13.61171600
H	1.56170200	3.23570900	14.42173500
C	1.54494900	2.65640600	12.42925800
C	1.20263800	1.33265000	12.12904200
C	1.66695200	0.66187800	11.02022200
H	1.44029000	-0.24264600	10.84179100
C	2.49629000	1.40760300	10.17621600
C	2.86311200	2.70849300	10.41837200
H	3.43308700	3.17218900	9.81652300
C	2.37951400	3.33607000	11.56967500
H	2.62735100	4.23424300	11.75943500
H	-0.00547200	0.12704000	13.21237100
Br	-4.84836400	-0.52747000	19.72240200
O	-1.05330700	-1.62992300	13.27468000
N	-2.04008500	-0.84989800	15.19918300
C	-1.80351200	-1.78491200	14.24896900
C	-2.59990000	-3.03371500	14.59733300
H	-2.00542000	-3.81755200	14.71062300
H	-3.27313200	-3.23570900	13.90060400
C	-3.25637900	-2.65640600	15.89308000
C	-2.91406800	-1.33265000	16.19329700
C	-3.37838300	-0.66187800	17.30211700
H	-3.15172000	0.24264600	17.48054800
C	-4.20772100	-1.40760300	18.14612300
C	-4.57454200	-2.70849300	17.90396700
H	-5.14451700	-3.17218900	18.50581600
C	-4.09094500	-3.33607000	16.75266300
H	-4.33878100	-4.23424300	16.56290400
H	-1.70595800	-0.12704000	15.10996800

2a-NHONHO

Atoms	x	y	z

Br	5.78439100	13.13693700	8.66107900
F	5.85219200	9.29780200	12.09078200
N	4.02482800	8.27702700	8.02812700
H	3.77131900	8.33276100	7.20761400
O	3.31724900	6.18298000	8.43842500
C	3.87710600	7.19500600	8.77730700
C	4.95207000	8.85157700	9.96339200
C	4.62772000	9.29780200	8.71289100
C	5.77348800	11.01270400	10.44930800
H	6.15769300	11.61065900	11.05019200
C	4.87426200	10.55918800	8.28999000
H	4.66225800	10.83815100	7.42910500
C	4.54106100	7.45165800	10.08102000
H	3.92437300	7.32710800	10.81948700
H	5.30728900	6.86930500	10.20553800
C	5.52077900	9.73527700	10.79799200
C	5.44683400	11.39330700	9.19180600
Br	0.31881900	1.44556300	5.34228200
F	0.25101800	5.28469800	1.91257900
N	2.07838200	6.30547300	5.97523400
H	2.33189100	6.24973900	6.79574700
O	2.78596100	8.39952000	5.56493600
C	2.22610500	7.38749400	5.22605400
C	1.15114000	5.73092300	4.03997000
C	1.47549100	5.28469800	5.29047000
C	0.32972200	3.56979600	3.55405300
H	-0.05448300	2.97184100	2.95316900
C	1.22894800	4.02331200	5.71337100
H	1.44095200	3.74434900	6.57425600
C	1.56214900	7.13084200	3.92234100
H	2.17883700	7.25539200	3.18387400
H	0.79592200	7.71319500	3.79782400
C	0.58243100	4.84722300	3.20536900
C	0.65637700	3.18919300	4.81155500

2b-NHONHO

Atoms	x	y	z

Br	2.31844000	8.96780000	12.74529900
F	2.07095900	12.43376900	8.99463900
O	-0.48835400	8.74337500	5.92482200
N	0.28980500	8.34556500	7.99171800
H	0.05569900	7.52044300	8.05095200
C	0.70868000	10.41773800	7.16271900
H	1.44694800	10.57776600	6.55466500
H	0.05788200	11.13091000	7.06814100
C	2.10147200	10.79250800	10.66215900
H	2.50612700	11.39448800	11.24411100
C	0.09219600	9.09510500	6.92046100
C	0.90931100	9.03677500	8.99435900
C	1.23246000	8.61825700	10.22665500
H	1.05934100	7.74895100	10.50710000
C	1.76808600	11.14832100	9.40465700
C	1.82451400	9.52674700	11.04445100
C	1.17831300	10.30399400	8.55605400
Br	-3.69542900	5.61470000	1.25806200
F	-3.44794900	2.14873100	5.00872200
O	-0.88863600	5.83912500	8.07853900
N	-1.66679400	6.23693500	6.01164300
H	-1.43268900	7.06205700	5.95240900
C	-2.08566900	4.16476200	6.84064200
H	-2.82393800	4.00473400	7.44869600
H	-1.43487200	3.45159000	6.93522100
C	-3.47846200	3.78999200	3.34120200
H	-3.88311700	3.18801200	2.75925000
C	-1.46918600	5.48739500	7.08290000
C	-2.28630100	5.54572500	5.00900200
C	-2.60945000	5.96424300	3.77670700
H	-2.43633100	6.83354900	3.49626100
C	-3.14507500	3.43417900	4.59870400
C	-3.20150400	5.05575300	2.95891000
C	-2.55530300	4.27850500	5.44730800

References

1. Lu, T.; Chen, F. Multiwfn: A multifunctional wavefunction analyzer. *J. Comput. Chem.* **2012**, *33*, 580–592, doi:10.1002/jcc.22885.

2. Frisch, M. J.; Trucks, G. W.; Schlegel, H. B.; Scuseria, G. E.; Robb, M. A.; Cheeseman, J. R.; Scalmani, G.; Barone, V.; Petersson, G. A.; Nakatsuji, H.; Li, X.; Caricato, M.; Marenich, A. V.; Bloino, J.; Janesko, B. G.; Gomperts, R.; Mennucci, B.; Hratch, W.C. Gaussian 16 2016, 2016.

3. Humphrey, W.; Dalke, A.; Schulten, K. VMD: Visual molecular dynamics. *J. Mol. Graph.* **1996**, *14*, 33–38, doi:10.1016/0263-7855(96)00018-5.

4. Silva, R.A.L.; de Brito, S.F.; Machado, D.F.S.; Carvalho-Silva, V.H.; de Oliveira, H.C.B.; Ribeiro, L. The influence of the configuration of the (C70)2 dimer on its rovibrational spectroscopic properties: a theoretical survey. *J. Mol. Model.* **2018**, *24*, 235, doi:10.1007/s00894-018-3780-y.

5. Chai, J.; Head-Gordon, M. Long-range corrected hybrid density functionals with damped atom–atom dispersion corrections. *Phys. Chem. Chem. Phys.* **2008**, *10*, 6615, doi:10.1039/b810189b.

6. Weigend, F.; Ahlrichs, R. Balanced basis sets of split valence, triple zeta valence and quadruple zeta valence quality for H to Rn: Design and assessment of accuracy. *Phys. Chem. Chem. Phys.* **2005**, *7*, 3297–3305, doi:10.1039/b508541a.

7. Bauzá, A.; Frontera, A. Halogen and Chalcogen Bond Energies Evaluated Using Electron Density Properties. *ChemPhysChem* **2020**, *21*, 26–31, doi:10.1002/cphc.201901001.

8. Bauzá, A.; Onero, D.Q.; Frontera, A. Substituent effects in multivalent halogen bonding complexes: A combined theoretical and crystallographic study. *Molecules* **2018**, *23*, doi:10.3390/molecules23010018.

9. Bartashevich, E. V; Tsirelson, V.G. Interplay between non-covalent interactions in complexes and crystals with halogen bonds. *Russ. Chem. Rev.* **2014**, *83*, 1181–1203, doi:10.1070/RCR4440.

10. Mata, I.; Alkorta, I.; Espinosa, E.; Molins, E. Relationships between interaction energy, intermolecular distance and electron density properties in hydrogen bonded complexes under external electric fields. *Chem. Phys. Lett.* **2011**, *507*, 185–189, doi:10.1016/j.cplett.2011.03.055.

11. Kuznetsov, M.L. Relationships between interaction energy and electron density properties for HoMO halogen bonds of the [(A)NY–X···X–Z(b)M] type (X = Cl, Br, I). *Molecules* **2019**, *24*, doi:10.3390/molecules24152733.
